# Long-term health outcomes of COVID-19 in ICU- and non-ICU-treated patients up to 2 years after hospitalization: a longitudinal cohort study (CO-FLOW)

**DOI:** 10.1186/s40560-024-00748-w

**Published:** 2024-11-08

**Authors:** J. C. Berentschot, L. M. Bek, M. H. Heijenbrok-Kal, J. van Bommel, G. M. Ribbers, J. G. J. V. Aerts, M. E. Hellemons, H. J. G. van den Berg-Emons, The CO-FLOW collaboration Group, Joachim G. J. V. Aerts, L. Martine Bek, Julia C. Berentschot, Rita J. G. van den Berg-Emons, Sieshem Bindraban, Wouter J. B. Blox, Jasper van Bommel, Shai A. Gajadin, Michel E. van Genderen, Diederik A. M. P. J. Gommers, Majanka H. Heijenbrok-Kal, Merel E. Hellemons, Roxane Heller, Erwin Ista, Stephanie van Loon-Kooij, Chantal Luijkx, Rutger Osterthun, Laurien Oswald, Gerard M. Ribbers, Ronald N. van Rossem, Herbert J. van de Sande, Robert van der Stoep, Janette J. Tazmi-Staal, Markus P. J. M. Wijffels, Eva G. Willems

**Affiliations:** 1 Department of Respiratory Medicine, Erasmus Medical Center, Rotterdam, The Netherlands; 2 Department of Rehabilitation Medicine, Erasmus Medical Center, Rotterdam, The Netherlands; 3 Rijndam Rehabilitation, Rotterdam, The Netherlands; 4 Department of Intensive Care, Erasmus Medical Center, Rotterdam, The Netherlands

**Keywords:** COVID-19, Long COVID, Intensive care, Long-term health outcomes

## Abstract

**Background:**

Many patients hospitalized for COVID-19 experience long-term health problems, but comprehensive longitudinal data up to 2 years remain limited. We aimed to (1) assess 2-year trajectories of health outcomes, including comparison between intensive care unit (ICU) treated and non-ICU-treated patients, and (2) identify risk factors for prominent health problems post-hospitalization for COVID-19.

**Methods:**

The CO-FLOW multicenter prospective cohort study followed adults hospitalized for COVID-19 at 3, 6, 12, and 24 months post-discharge. Measurements included patient-reported outcomes (a.o., recovery, symptoms, fatigue, mental health, sleep quality, return to work, health-related quality of life [HRQoL]), and objective cognitive and physical tests. Additionally, routine follow-up data were collected.

**Results:**

650 patients (median age 60.0 [IQR 53.0–67.0] years; 449/650 [69%] male) surviving hospitalization for COVID-19 were included, of whom 273/650 (42%) received ICU treatment. Overall, outcomes improved over time. Nonetheless, 73% (322/443) of patients had not completely recovered from COVID-19, with memory problems (274/443; 55%), concentration problems (259/443; 52%), and dyspnea (251/493; 51%) among most frequently reported symptoms at 2 years. Moreover, 61% (259/427) had poor sleep quality, 51% (222/433) fatigue, 23% (102/438) cognitive failures, and 30% (65/216) did not fully return to work. Objective outcome measures showed generally good physical recovery. Most outcomes were comparable between ICU- and non-ICU-treated patients at 2 years. However, ICU-treated patients tended to show slower recovery in neurocognitive symptoms, mental health outcomes, and resuming work than non-ICU-treated patients, while showing more improvements in physical outcomes. Particularly, female sex and/or pre-existing pulmonary disease were major risk factors for poorer outcomes.

**Conclusions:**

73% (322/443) of patients had not completely recovered from COVID-19 by 2 years. Despite good physical recovery, long-term neurocognitive complaints, dyspnea, fatigue, and impaired sleep quality persisted. ICU-treated patients showed slower recovery in neurocognitive and mental health outcomes and resumption of work. Tailoring long-term COVID-19 aftercare to individual residual needs is essential. Follow-up is required to monitor further recovery.

*Trial registration*: NL8710, registration date 12-06-2020.

**Supplementary Information:**

The online version contains supplementary material available at 10.1186/s40560-024-00748-w.

## Introduction

More than 3 years after the onset of the COVID-19 pandemic, over 771 million people worldwide have been infected with SARS-CoV-2 [1]. Although a large proportion of infections has a mild disease course, hospitalization including intensive care unit (ICU) admission for respiratory failure may be required. Many patients do not fully recover to their pre-COVID-19 health status after hospitalization [2], experiencing a wide range of persistent health problems with fatigue and neurocognitive problems among the most frequently reported [3, 4]. Furthermore, incomplete recovery after COVID-19 infection is associated with reduced health-related quality of life (HRQoL) [4, 5]. Patients with COVID-19 who suffer persistent health problems place a considerable strain on healthcare services and medical costs, on top of the personal and societal impacts [6].

Although several studies report health problems after COVID-19 up to one year after hospitalization [3, 4, 7, 8], data beyond one year remain limited. Two large cohort studies from Wuhan, China, showed that while the proportion of patients with persisting symptoms decreased over time, the majority still experienced symptoms 2 years after hospitalization for COVID-19 [4, 9]. Also population-based studies involving non-hospitalized individuals showed persisting symptoms up to 2 years, with more severely affected individuals having an increased risk of symptom manifestations [10, 11]. After ICU treatment, patients often experience persistent symptoms, including physical, cognitive, and mental problems, generally referred to as the Post-Intensive Care Syndrome (PICS) [12]. In the Wuhan studies, only 4% (51/1192) [4] and 1.9% (36/1864) [9] of the patients required ICU treatment for COVID-19, limiting inferences about different aftercare needs for ICU- and non-ICU-treated patients. One European study found that 84% of their patients experienced symptoms affecting daily life 2 years after hospitalization for COVID-19, with comparable prevalence of symptoms in ICU- and non-ICU-treated patients [13]. While this finding is in line with several short-term studies [14, 15], others have reported more sequelae in ICU-treated patients compared with non-ICU-treated patients [5, 16, 17]. Overall, a more comprehensive and multidimensional longitudinal evaluation of long-term health outcomes beyond one year and identification of patients at risk for poor outcomes after hospitalization for COVID-19 are pivotal for refining aftercare strategies. Moreover, an evaluation on potential disparities in long-term health outcomes between ICU- and non-ICU-treated patients with COVID-19 is required. Our study is particularly well-suited for comparing ICU-treated and non-ICU-treated patients, as our study contains a higher proportion of ICU patients compared to the average proportion of ICU admissions across all Dutch hospitals [18].

Our primary aim of this study was to assess trajectories of a comprehensive range of health outcomes, both patient-reported and objectively measured, in patients with COVID-19 up to 2 years after hospital discharge, including a comparison between ICU- and non-ICU-treated patients. The secondary aim was to identify risk factors for self-reported recovery status and prominent long-term health problems in these patients: fatigue, cognitive failures, sleep quality, and health-related quality of life.

## Methods

### Study design and participants

We performed a 2-year prospective multicenter cohort study, COvid-19 Follow-up care paths and Long-term Outcomes Within the Dutch health care system (CO-FLOW), that follows up patients discharged from hospitals in the Rotterdam–Rijnmond–Delft region in the Netherlands. This study was performed in 7 hospitals (1 academic and 6 regional hospitals) and 3 rehabilitation centers (1 medical rehabilitation center and 2 skilled nursing facilities). This study included patients between July 2020 and October 2021 who had been hospitalized for COVID-19 (diagnosis by laboratory or clinical findings), aged ≥ 18 years, had sufficient knowledge of the Dutch or English language, and were within 6 months post-discharge. Incapacitated patients (e.g., dementia) were not included. Eligible patients were informed about the CO-FLOW study at hospital discharge and were recruited during routine follow-up at the outpatient clinic of one of the participating centers or during their inpatient stay in a rehabilitation center. In the Netherlands, it is standard practice to offer post-discharge follow-up to patients with COVID-19 at the outpatient clinic of the discharging hospital, with the first visit generally scheduled 6–12 weeks post-discharge. Recruitment of study participants occurred independently of the patient’s recovery status and primarily depended on availability of research personnel. The CO-FLOW study protocol has been described in detail elsewhere [19]. The Medical Ethics Committee of the Erasmus MC, University Medical Center Rotterdam, approved this study (MEC-2020-0487). This study has been prospectively registered in the International Clinical Trial Registry Platform (NL8710). Participants provided written informed consent before the start of study measurements. We reported this observational study according to STROBE guidelines.

### Procedures

Study visits were performed at 3, 6, 12, and 24 months after hospital discharge at the outpatient clinic of one of the participating hospitals. For patients unable to visit the hospital for study visits, a research assistant performed study visits at home. During study visits, physical and cognitive tests and recovery and symptom checklist were administered. In addition, a survey of validated patient-reported outcome measures (PROMs) was sent via e-mail or postal mail. Baseline characteristics and routine follow-up data regarding pulmonary and radiological sequelae were retrospectively collected from medical records at the participating facilities and during the first study visit. We collected patients’ age, sex, body mass index (BMI), migration background, education level, employment status, smoking status, pre-COVID-19 leisure time physical activity, assessed with the Saltin–Grimby Physical Activity Level Scale questionnaire [20], and comorbidities at hospital admission. In-hospital characteristics included COVID-19 wave, the first assessment upon admission of laboratory values and chest X-ray abnormalities, type of treatment for COVID-19, thrombosis, delirium, maximum level and type of oxygen support, ICU treatment, length of stay (LOS) in ICU, and LOS in hospital. Additionally, we collected information on patient discharge destination after hospitalization. Routine follow-up at hospitals generally took place around 6 weeks to 3 months post-discharge and were generally continued around 6, 12, and 24 months if residual pulmonary abnormalities persisted. All collected data were stored in the Castor Electronic Data Capture system (Castor EDC, Amsterdam, the Netherlands).

### Study outcome measurements

#### Recovery and symptoms

Self-reported recovery status from COVID-19, as compared to pre-COVID-19 health status, was assessed with the Core Outcome Measure for self-reported recovery from COVID-19 and dichotomized into completely recovered and not completely recovered (mostly recovered, somewhat recovered, half recovered, and not recovered at all) [21]. New symptoms since COVID-19 were assessed using a symptom questionnaire (Corona Symptom Checklist, 26 symptoms) to assess the presence of new or worsened symptoms following SARS-CoV-2 infection. At the 24-month visit, patients were asked to also rate the severity (mild, moderate, severe, or very severe) of these symptoms.

#### PROMs

Fatigue was assessed with the Fatigue Assessment Scale (scores 0–50, cutoff ≥ 22) [22]; dyspnea with the Modified Medical Research Council Dyspnea Scale [23, 24]; anxiety and depression with the Hospital Anxiety and Depression Scale, subscales Anxiety and Depression (subscale scores 0–21, cutoff ≥ 11) [25]; posttraumatic stress disorder (PTSD) with the Impact of Event Scale-Revised (scores 0–88, cutoff ≥ 33) [26, 27]; cognitive failures with the Cognitive Failures Questionnaire (CFQ, scores 0–100, cutoff > 43) [28, 29]; sleep quality with the Pittsburgh Sleep Quality Index (scores 0–21, cutoff ≥ 5) [30]; independency in activities of daily life with the Barthel Index (scores 0–20) [31]; physical fitness with the International Fitness Scale (scored as very poor, poor, average, good, or very good) [32]; physical activity with the International Physical Activity Questionnaire (expressed in MET-minutes/week) [33]; participation in daily life activities with the Utrecht Scale for Evaluation of Rehabilitation-Participation on three scales: frequency, restrictions, and satisfaction (subscale scores 0–100) [34]; employment status with the iMTA Productivity Cost Questionnaire (categorized into no, partial, or full return to work) for patients with a paid job before SARS-CoV-2 infection [35]; and health-related quality of life with the 5-level EuroQoL-5D (EQ-5D-5L) questionnaire and the 36-item Short Form Health Survey (SF-36). The EQ-5D-5L consists of the 5-level EQ-5D index value (0 indicates death and 1 perfect health; negative scores indicate a health status worse than death) and a visual analogue scale (EQ-VAS, scores 0–100) [36]. The SF-36 consists of 8 domains (scores 0–100) and a physical and mental component summary score [37].

#### Objective study measurements

Cognitive functioning was assessed with the Montreal Cognitive Assessment (MoCA) (score range 0–30, cutoff < 26) [38] at the patient’s first study visit, and, only if score < 26, repeated at subsequent visits. Physical function was evaluated for aerobic capacity with the 6 min walk test (6MWT) assessing the 6 min walk distance (6MWD) [39] and the 1 min sit-to-stand test (1MSTST) assessing the number of sit-to-stand repetitions [40]. Muscle strength was assessed by measurement of maximum isometric handgrip strength (HGS) in kg over three attempts per hand [41]. Mobility was assessed with the De Morton Mobility Index (DEMMI) test (scores 0–100) [42, 43]. Outcomes of the 6MWT [44], 1MSTST [45], and HGS [46] were normalized to the percentage of normative values using reference values, as well as to performance below the lower limit of normal (LLN) for the 6MWT.

#### Routine follow-up data

Pulmonary function tests (PFT) consisted of spirometry measuring forced vital capacity (FVC) and forced expiratory volume in 1 s (FEV_1_) in liters, and diffusion capacity of the lungs for carbon monoxide adjusted for hemoglobin (DLCOc) in mmol min^−1^ kPa^−1^, according to the standards of the American Thoracic Society and European Respiratory Society [47]. PFT parameters are also expressed as a percentage of predicted FVC, FEV_1_, and DLCOc values, using references values from the Global Lung Function Initiative Network [48, 49]. A value below the LLN (*z*-score < − 1.64) was defined as abnormal. Radiographic evaluation consisted of chest radiography or thin-section non-contrast chest-CT scan, which was interpreted by experienced radiologists using a standardized assessment. Chest radiographs were classified as normal, moderate, or severe abnormalities. CT scans were scored for the presence of abnormalities including ground-glass opacities (none, moderate, or severe), bronchiectasis or bronchiolectasis (none, moderate, or severe), consolidations, reticulation/fibrosis, and subpleural lines and bands.

### Statistical analysis

Data are presented as mean with standard deviation (SD) and/or median with interquartile range (IQR) or as number with percentage. Group comparisons (ICU vs. non-ICU) were performed for continuous variables with the Mann–Whitney *U* test and for categorical variables with the Chi-squared test. For cognitive function, scores ≥ 26 were carried forward in future study visits. For the primary aim, we used Generalized Estimating Equations (GEE) with repeated measurements to explore the trajectories of health outcomes over time in the total cohort and comparing ICU and non-ICU groups. The GEE is a semi-parametric approach which considers within- and between-subject correlations and uses all available measurements despite incomplete data. We entered follow-up time (3, 6, 12, and 24 months) as a fixed factor in the GEE analysis for the total cohort. Additionally, we entered group (ICU vs. non-ICU) as a fixed factor and the interaction of follow-up time with group in the GEE for the subgroup analyses. The GEE outcomes of the 2-year trajectories for physical (percentage of normative values) and mental health outcomes are displayed graphically; for mental health variables the GEE analysis was adjusted for age and sex. For the secondary aim, we used GEE analyses to assess risk factors for recovery status, fatigue, cognitive failures, sleep quality, and HRQoL over the 2-year follow-up period. We selected covariables (i.e., characteristics at hospital admission) a priori and entered them as fixed factors in each GEE analysis, including time (follow-up visits), age, sex (male or female), obesity (obese if BMI ≥ 30 kg/m^2^, yes/no), cardiovascular disease (yes/no), pulmonary disease (yes/no), diabetes (yes/no), migration background (European or non-European), education (low, middle, or high), employment status (employed, unemployed, or retired), smoking status (current/former or never), steroid or anti-inflammatory treatment (yes/no), ICU admission (yes/no), and the LOS in hospital (days). Each GEE analysis was performed using an unstructured correlation matrix, without data imputation. A P value below 0.05 was considered statistically significant, unless stated otherwise. We used a Bonferroni-corrected α threshold to correct for multiple comparisons in recovery and symptoms (α = 0.00185), validated PROMs (α = 0.00417), objective study measurements (α = 0.01), and routine follow-up data (α = 0.00556). All statistical analyses were performed with IBM SPSS Statistics version 28 (SPSS Inc., Chicago, IL, USA).

## Results

**Fig. 1:**
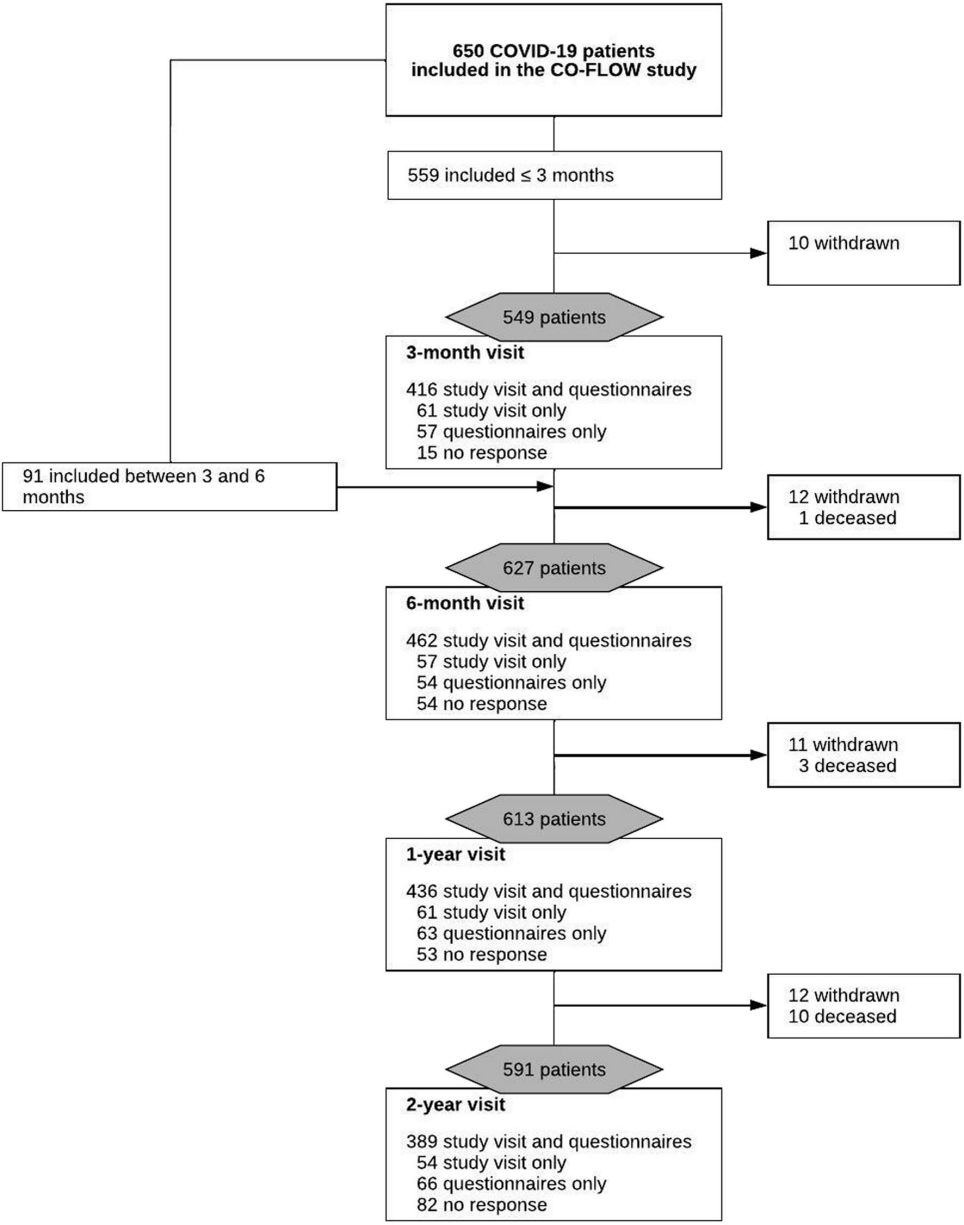
Flowchart of CO-FLOW study visits

We included 650 patients after hospitalization for COVID-19 (Fig. 1), all discharged between March 24, 2020 and June 17, 2021; 273 (42%) patients received ICU treatment. Study visits were performed between July 1, 2020 and June 7, 2023. Table 1 shows the baseline characteristics at hospital admission. For the total cohort, the median age was 60.0 (53.0–67.0) years and 449 (69%) were male. Compared to the non-ICU group, the ICU group comprised more males (205 [75%] vs. 244 [65%], *p* = 0.005) and non-Europeans (95 [36%] vs. 86 [23%], *p* < 0.001), and more frequently had obesity (145 [53%] vs. 121 [32%], *p* < 0.001). Most patients in the ICU group (235 [86%]) required invasive mechanical ventilation for a median duration of 15.0 (8.5–28.0) days and patients had longer overall median LOS in hospital than the non-ICU group (31.0 [19.0–47.0] vs. 6.0 [4.0–10.5] days, *p* < 0.001). Moreover, ICU-treated patients were more frequently discharged to inpatient rehabilitation, whereas non-ICU-treated patients were mostly discharged home after hospitalization.

**Table 1:** Baseline characteristics of study participants

	*N*^a^	All (*N* = 650)	Non-ICU (*n* = 377)	ICU (*n* = 273)	*P* value
**Patient characteristics**					
*Age, years*					0.19
Mean		59.7 (11.4)	60.6 (11.4)	58.6 (11.5)	
Median		60.0 (53.0–67.0)	61.0 (53.0–68.0)	60.0 (53.0–67.0)	
*Sex, male*		449 (69%)	244 (65%)	205 (75%)	**0.005**
*BMI, kg/m*^*2*^					** < 0.001**
Mean	589	29.4 (5.4)	28.5 (5.1)	30.5 (5.5)	
Median		28.4 (25.7–32.2)	27.6 (25.3–31.0)	29.7 (26.3–33.3)	
*Migration background*	630				** < 0.001**
European		449 (71%)	280 (77%)	169 (64%)	
Dutch Caribbean		89 (14%)	42 (11%)	47 (18%)	
Asian		39 (6%)	19 (5%)	20 (7%)	
Turkish		27 (5%)	13 (4%)	15 (6%)	
(North) African		25 (4%)	12 (3%)	13 (5%)	
*Education*^b^	625				0.40
Low		222 (35%)	130 (36%)	92 (35.5%)	
Middle		218 (35%)	121 (33%)	97 (37.5%)	
High		185 (30%)	115 (31%)	70 (27%)	
*Employment*	627				0.28
Unemployed		100 (16%)	60 (16%)	40 (15%)	
Employed		372 (59%)	208 (57%)	164 (63%)	
Retired		155 (25%)	98 (27%)	57 (22%)	
*Smoking status*	631				0.53
Never		280 (44%)	159 (43%)	121 (46%)	
Former		339 (54%)	199 (54%)	140 (53%)	
Current		12 (2%)	9 (3%)	3 (1%)	
*Physical activity level*^c^	624				0.07
Inactive		86 (14%)	61 (17%)	25 (10%)	
Light		332 (53%)	186 (51%)	146 (56%)	
Moderate		168 (27%)	94 (26%)	74 (29%)	
Vigorous		38 (6%)	24 (7%)	14 (5%)	
*Comorbidities*					
≥ 1		543 (83%)	303 (82%)	231 (85%)	0.21
Obesity (BMI ≥ 30 kg/m^2^)		266 (41%)	121 (32%)	145 (53%)	** < 0.001**
Diabetes		130 (20%)	78 (21%)	52 (19%)	0.61
Cardiovascular disease/hypertension		257 (40%)	146 (39%)	111 (41%)	0.62
Pulmonary disease		162 (25%)	101 (27%)	61 (22%)	0.20
Renal disease		59 (9%)	38 (10%)	21 (8%)	0.30
Gastrointestinal disease		31 (5%)	20 (5%)	11 (4%)	0.45
Neuromuscular disease		68 (11%)	42 (11%)	26 (10%)	0.51
Malignancy		69 (11%)	40 (11%)	29 (11%)	1.00
Autoimmune/inflammatory disease		68 (11%)	48 (13%)	20 (7%)	**0.03**
Mental disorder		32 (5%)	21 (6%)	11 (4%)	0.37
*Vaccinated before admission*	641				NA
Yes		5 (1%)	3 (1%)	2 (1%)	
No		636 (99%)	368 (99%)	268 (99%)	
**In-hospital characteristics**					
*COVID-19 wave*^*d*^					** < 0.001**
First		180 (28%)	72 (19%)	108 (40%)	
Second		339 (52%)	224 (59%)	115 (42%)	
Third		131 (20%)	81 (22%)	50 (18%)	
*Laboratory values*					
Creatinine, µmol/L	618	83.0 (69.8–101.3)	81.0 (68.0–95.3)	87.0 (72.0–109.8)	** < 0.001**
(CKD-EPI) eGFR, ml/min	603	82.0 (66.0–90.0)	83.5 (68.0–90.0)	80.0 (62.5–90.0)	0.07
CRP, mg/L	614	89.0 (48.0–154.3)	74.0 (41.0–121.0)	127.0 (67.0–193.0)	** < 0.001**
Ferritin, µg/L	376	833.5 (453.3–1592.3)	665.0 (368.0–1221.0)	1170.0 (585.0–2010.5)	** < 0.001**
ALAT, U/L	598	37.0 (25.0–56.0)	35.5 (24.0–53.0)	40.0 (27.8–62.0)	**0.02**
Hemoglobin, mmol/L	616	8.6 (7.9–9.2)	8.6 (7.9–9.2)	8.5 (7.8–9.2)	0.33
MCV, fl	604	88.0 (85.0–91.0)	88.0 (85.0–91.0)	88.0 (85.0–91.0)	0.60
Trombocyten, 10^3^/L	608	211.0 (160.0–276.0)	210.0 (161.0–273.3)	213.0 (160.0–284.0)	0.44
Lymphocytes absolute count, 10^3^/L	432	0.9 (0.7–1.2)	0.9 (0.7–1.2)	0.9 (0.6–1.1)	0.09
D-dimer, mg/L	313	1.1 (0.6–35.2)	1.2 (0.7–708.3)	1.0 (0.6–3.7)	**0.003**
NT-pro-BNP, pmol/ml	118	18.0 (8.0–53.0)	18.0 (7.0–76.0)	21.0 (8.0–45.0)	0.78
IL-6, pmol/ml	47	53.0 (26.0–173.0)	28.5 (24.5–45.0)	88.0 (28.0–213.5)	**0.03**
*Chest x-ray abnormalities*	619				** < 0.001**
Normal		67 (11%)	55 (15%)	12 (5%)	
Moderate		135 (22%)	98 (27%)	37 (15%)	
Severe		417 (67%)	213 (58%)	204 (81%)	
*COVID-19 directed treatment*					0.36
None		148 (23%)	81 (22%)	67 (25%)	
(Hydroxy)chloroquine		12 (2%)	3 (1%)	9 (3%)	
Steroids		456 (70%)	275 (73%)	181 (66%)	
Antivirals		97 (15%)	76 (20%)	21 (8%)	
Anti-inflammatory		76 (12%)	11 (3%)	65 (24%)	
Convalescent plasma		8 (1%)	6 (1%)	2 (1%)	
Thrombosis	648	102 (16%)	19 (5%)	83 (31%)	** < 0.001**
Delirium	648	165 (26%)	14 (4%)	151 (56%)	** < 0.001**
Requiring oxygen supplementation		627 (97%)	354 (94%)	273 (100%)	** < 0.001**
Requiring high flow nasal cannula	648	208 (32%)	57 (15%)	151 (56%)	** < 0.001**
ICU admission		273 (42%)	–	273 (42%)	NA
Invasive mechanical ventilation		235 (36%)	–	235 (86%)	NA
*Length of intubation, days*	229		–		NA
Mean		20.1 (15.4)		20.1 (15.4)	
Median		15.0 (8.5–28.0)		15.0 (8.5–28.0)	
Tracheostomy	648	90 (14%)	–	90 (33%)	NA
*Length of ICU stay, days*	271		–		NA
Mean		22.0 (17.5)		22.0 (17.5)	
Median		16.0 (9.0–31.0)		16.0 (9.0–31.0)	
*Length of hospital stay, days*					** < 0.001**
Mean		19.7 (20.2)	8.5 (7.4)	35.2 (21.9)	
Median		12.0 (6.0–28.0)	6.0 (4.0–10.5)	31.0 (19.0–47.0)	
*Discharge destination*					** < 0.001**
Home		481 (74%)	354 (94%)	127 (46%)	
Inpatient medical rehabilitation center		80 (12%)	2 (1%)	78 (29%)	
Inpatient skilled nursing facility		89 (14%)	21 (5%)	68 (25%)	
*Time interval from discharge to follow-up visits, days*					
3 months	430	93.0 (88.0–103.0)	93.0 (87.0–101.0)	93.0 (88.0–105.8)	0.14
6 months	517	184.0 (180.0–193.0)	185.0 (180.0–193.0)	183.0 (178.8–193.0)	0.07
1 year	502	366.0 (361.0–373.0)	366.0 (361.0–373.0)	365.5 (362.0–372.0)	0.59
2 years	449	730.0 (725.0–737.5)	731.0 (725.0–739.0)	729.0 (725.3–735.0)	**0.04**

Data are presented as mean (standard deviation), median (interquartile range), or *n* (%). Patient characteristics are presented for pre-COVID-19 situation, and age and BMI at the time of hospital admission. The following variables were dichotomized for statistical analysis, migration background was categorized as European versus non-European groups combined, smoking status as never versus former/current, and treatment as no treatment versus any received treatment. *P* values are obtained using Mann–Whitney U test, or Chi-squared test as appropriate; a *P* value less than 0.05 was considered statistically significant and is indicated in bold

*BMI* Body Mass Index, *ICU* Intensive Care Unit, *NA* not applicable

^a^Adjusted n is presented for variables with a total number of patients less than 650

^b^Education comprises low (primary or secondary education); middle (high school); high (postsecondary education or university)

^c^Pre-COVID-19 leisure time physical activity level was measured with the Saltin–Grimby Physical Activity Level Scale questionnaire [20]

^d^We classified patients by discharge date: the first COVID-19 wave (Feb–Jun 2020; original variant dominant), second wave (Jul 2020–Feb 2021; alpha variant dominant), and third wave (Feb-Jun 2021; beta and delta variants dominant)

### Recovery status and symptoms

#### Total cohort

Recovery status, having ≥ 1 symptom, and proportion of symptoms of impaired fitness, fatigue, dyspnea, muscle weakness, hair loss, sleep disturbances, and joint pain improved significantly over 2 years in the total cohort, whereas proportion of hearing problems worsened (all *p* < 0.00185) (Table 2 and Supplementary Table S1). At 2 years, 73% (322/443) of patients had not completely recovered from COVID-19. Regarding symptoms, 88% (443/503) experienced ≥ 1 symptoms, most frequently impaired fitness (62%), fatigue (61%), memory problems (55%), concentration problems (52%), and dyspnea (51%). Patients indicated these symptoms as severe or very severe for impaired fitness in 33% (85/254), fatigue in 43% (108/253), memory problems in 36% (82/225), and concentration problems in 37% (79/217) (Supplementary Table S2).

**Table 2:** Trajectories of self-reported recovery and the ten most prevalent symptoms in ICU- and non-ICU-treated patients for COVID-19 up to 2 years after hospital discharge

	Total					Non-ICU				ICU				Overall comparison, ICU vs. non-ICU	Interaction ICU * Time
	3 M	6 M	1 Y	2 Y	P value	3 M	6 M	1 Y	2 Y	3 M	6 M	1 Y	2 Y	*P* value	*P* value
*Recovery status, n*	*159*	*300*	*418*	*443*		*90*	*184*	*225*	*260*	*69*	*116*	*193*	*183*		
Not completely recovered	142 (89%)	248 (83%)	316 (77%)	322 (73%)	** < 0.001**	78 (87%)	142 (77%)	159 (74%)	180 (69%)	64 (93%)	106 (91%)	157 (81%)	142 (78%)	0.003	0.08
*Symptoms, n*	*441*	*528*	*532*	*503*		*275*	*311*	*310*	*300*	*166*	*218*	*222*	*203*		
Impaired fitness	362 (83%)	379 (72%)*	346 (65%)*^#^	311 (62%)*^†^	** < 0.001**	217 (79%)	218 (70%)	196 (63%)	165 (58%)	145 (88%)	161 (74%)	149 (67%)	138 (68%)	0.02	0.17
Fatigue	116/140 (83%)	162/241 (67%)*	237/380 (62%)^#^	302/493 (61%)	** < 0.001**	74/84 (88%)	93/140 (66%)	146/235 (62%)	190/292 (58%)	42/56 (75%)	69/101 (68%)	91/145 (63%)	132/201 (66%)	0.99	0.16
Dyspnea	87/128 (68)%	127/237 (54%)*	210/378 (56%)	251/493 (51%)	**0.001**	61/78 (78%)	74/135 (55%)	133/235 (56%)	139/292 (48%)	26/50 (52%)	53/102 (52%)	77/143 (54%)	112/201 (56%)	0.47	0.005
Muscle weakness	253 (58%)	258 (49%)*	225 (42%)*^#^	189 (38%)^†^	** < 0.001**	143 (52%)	135 (43%)	116 (38%)	105 (35%)	110 (66%)	123 (57%)	108 (49%)	84 (41%)	** < 0.001**	0.23
Memory problems	238 (54%)	302 (57%)	296 (56%)	274 (55%)	0.44	163 (59%)	190 (61%)	177 (57%)	155 (52%)	75 (45%)	112 (52%)	119 (54%)	119 (59%)	0.11	**0.001**
Concentration problems	232 (53%)	273 (52%)	271 (51%)	259 (52%)	0.81	158 (58%)	166 (54%)	159 (51%)	150 (50%)	74 (45%)	107 (49%)	111 (50%)	109 (54%)	0.19	0.03
Sensory overload	50/109 (46%)	100/229(44%)	145/381 (38%)	196/495 (40%)	0.50	33/61 (54%)	56/129 (43%)	90/236 (38%)	103/294 (35%)	17/48 (35%)	44/100 (44%)	55/145 (38%)	90/201 (46%)	0.65	0.09
Joint pain	187 (43%)	218 (41%)	217 (41%)	170 (34%)^†^	** < 0.001**	109 (40%)	113 (36%)	110 (36%)	92 (31%)	78 (48%)	105 (48%)	107 (48%)	78 (38%)	0.002	0.75
Balance problems/dizziness	184 (42%)	228 (44%)	223 (42%)	200 (40%)	0.53	118 (43%)	126 (41%)	123 (40%)	104 (35%)	66 (40%)	102 (47%)	99 (45%)	96 (48%)	0.09	0.09
Sleep disturbances	160 (36%)	182 (35%)	185 (35%)	141 (28%)*	**0.002**	108 (39%)	119 (39%)	101 (33%)	73 (24%)	52 (31%)	63 (29%)	83 (37%)	68 (34%)	0.97	** < 0.001**

The data comprise raw test outcomes and are presented as n (%) or as *n*/*N* (%) in the case of adjusted total number. Recovery status from COVID-19 was dichotomized into completely recovered and not complete recovered (not recovered at all, somewhat recovered, half recovered, or mostly recovered). The presence of symptoms was assessed with a symptom questionnaire (Corona Symptom Checklist, CSC) on new or worsened symptoms following SARS-CoV-2 infection. The symptoms fatigue, dyspnea, and sensory overload were added to the CSC in a later stage and therefore contain lower total numbers. The trajectories of all the assessed symptoms are shown in Supplementary Table S1. *P* values are obtained from Generalized Estimating Equations analysis, a *P* value less than 0.00185 was considered statistically significant and is indicated in bold. In the total cohort, *indicates a significant difference as compared to the previous study visit, ^#^indicates a significant difference between the 3-month and 1-year study visits, and ^†^between the 6-month and 2-year study visits. ^†^indicates significant difference between ICU and non-ICU group at 2 years

#### ICU- vs. non-ICU-treated patients

On average, the proportion of patients with muscle weakness, tingling/numbness in extremities, and hoarseness was significantly higher in the ICU group than in the non-ICU group (all *p* < 0.001); other symptoms were comparable (Table 2). Over time, the ICU group was more likely to experience memory problems (OR 2.1 [95%CI 1.4–3.1], *p* < 0.001) and sleep disturbances (2.2 [1.4–3.4], *p* < 0.001) compared to the non-ICU group. At 2 years, outcomes did not differ significantly between groups, except a higher proportion of hoarseness in the ICU group (*p* < 0.001).

### PROMs

#### Total cohort

Outcomes of fatigue, mental health, sleep quality, physical fitness, participation, return to work, and HRQoL improved significantly (all *p* < 0.00417) over time in the total cohort (Table 3). At 2 years, 51% (222/433) of patients experienced fatigue, 10% (43/446) anxiety, 10% (45/446) depression, 7% (31/446) PTSD, 23% (102/446) cognitive failures, 61% (259/427) poor sleep quality, 18% (81/447) poor or very poor physical fitness, and, among patients with a paid job before COVID-19, 30% (65/216) had not fully returned to work. Regarding HRQoL, patients reached a mean EQ-5D index value of 0.80 (0.22) and EQ-VAS of 73.4 (18.2) by 2 years.

**Table 3:** Trajectories of validated PROMs in COVID-19 patients up to 2 years after hospital discharge

	Total					Non-ICU				ICU				Overall comparison, ICU vs. non-ICU	Interaction ICU * time
	3 M	6 M	1 Y	2 Y	*p* value	3 M	6 M	1 Y	2 Y	3 M	6 M	1 Y	2 Y	*P* value	*P* value
*Fatigue, n*	*438*	*483*	*478*	*433*		*272*	*289*	*288*	*257*	*166*	*194*	*190*	*176*		
FAS, total score	25.1 (9.4)	24.2 (9.1)*	23.7 (8.9)*^#^	23.6 (8.9)^†^	** < 0.001**	25.6 (9.7)	24.7 (9.4)	24.0 (8.9)	23.5 (9.0)	24.5 (8.9)	23.5 (8.6)	23.2 (8.9)	23.7 (8.9)	0.45	0.31
Fatigue (FAS ≥ 22)	258 (59%)	278 (58%)	266 (56%)	222 (51%)^†^	**0.004**	166 (61%)	170 (59%)	162 (56%)	132 (51%)	92 (55%)	108 (56%)	103 (54%)	90 (51%)	0.64	0.86
*mMRC dyspnea scale, n*	*433*	*484*	*473*	*466*		*270*	*289*	*285*	*278*	*163*	*195*	*188*	*188*		
mMRC ≥ 1	175 (40%)	174 (36%)	163 (35%)	187 (40%)	0.08	117 (43%)	110 (38%)	107 (38%)	113 (41%)	58 (36%)	64 (33%)	56 (30%)	74 (40%)	0.15	0.60
*Mental health, n*	*436*	*486*	*483*	*446*		*274*	*294*	*291*	*263*	*163*	*194*	*194*	*183*		
HADS-A, total score	5.3 (4.3)	4.7 (4.2)*	4.8 (4.5)^#^	4.6 (4.2)	** < 0.001**	5.5 (4.3)	4.9 (4.3)	4.8 (4.3)	4.5 (4.7)	5.0 (4.2)	4.4 (4.0)	4.8 (4.7)	4.7 (4.3)	0.64	0.23
Anxiety (HADS-A ≥ 11)	56 (13%)	50 (10%)	53 (11%)	43 (10%)	0.27	38 (14%)	31 (11%)	28 (10%)	19 (7%)	18 (11%)	19 (10%)	25 (13%)	24 (13%)	0.49	0.01
HADS-D, total score	5.0 (4.1)	4.5 (4.0)*	4.4 (4.1)^#^	4.5 (3.9)	**0.004**	5.2 (4.1)	4.7 (4.0)	4.7 (3.8)	4.5 (3.8)	4.5 (4.2)	4.1 (4.0)	4.0 (4.3)	4.6 (4.1)	0.31	0.13
Depression (HADS-D ≥ 11)	49 (11%)	45 (9%)	50 (10%)	45 (10%)	0.84	34 (13%)	33 (11%)	28 (10%)	23 (9%)	15 (9%)	12 (6%)	22 (12%)	22 (12%)	0.99	0.004
IES-R, total score	14.1 (13.9)	12.2 (12.6)*	12.0 (12.5)^#^	10.9 (12.5)*^†^	** < 0.001**	13.4 (13.2)	11.7 (12.2)	10.7 (11.6)	9.2 (11.5)	15.5 (14.9)	12.9 (13.2)	13.9 (13.6)	13.2 (13.4)	0.005	**0.002**
PTSD (IES-R ≥ 33)	51 (12%)	41 (9%)	35 (7%)^#^	31 (7%)	**0.004**	28 (10%)	22 (8%)	15 (5%)	12 (5%)	23 (14%)	19 (10%)	20 (10%)	19 (11%)	0.01	0.06
*Cognition, n*	*433*	*476*	*470*	*438*		*271*	*290*	*282*	*259*	*162*	*186*	*188*	*179*		
CFQ, total score	29.7 (19.2)	29.4 (18.5)	30.7 (18.7)	30.4 (18.3)	0.16	32.0 (19.7)	30.5 (19.0)	31.8 (18.7)	30.4 (18.3)	25.9 (17.9)	27.9 (17.6)	29.1 (18.8)	30.6 (18.4)	0.10	0.007
Cognitive failure (CFQ > 43)	95 (22%)	114 (24%)	103 (22%)	102 (23%)	0.31	67 (25%)	74 (26%)	66 (23%)	58 (22%)	28 (17%)	40 (22%)	37 (20%)	44 (25%)	0.30	0.27
*Sleep quality, n*	*428*	*471*	*462*	*427*		*264*	*284*	*282*	*251*	*164*	*187*	*180*	*176*		
PSQI, total score	7.0 (4.2)	6.8 (4.2)	6.5 (4.1)^#^	6.5 (4.3)	**0.002**	7.3 (4.3)	6.9 (4.4)	6. (4.0)	6.6 (4.2)	6.5 (3.9)	6.4 (3.8)	6.5 (4.3)	6.3 (4.4)	0.38	0.06
PSQI, Poor sleep quality (PSQI ≥ 5)	286 (67%)	311 (66%)	275 (60%)^#^	259 (61%)	**0.001**	179 (68%)	189 (67%)	164 (58%)	157 (63%)	107 (65%)	122 (65%)	111 (62%)	102 (58%)	0.81	0.21
*Functioning in ADL, n*	*448*	*499*	*491*	*449*		*271*	*302*	*297*	*263*	*177*	*197*	*194*	*186*		
Barthel index, total score	19.5 (1.5)	19.5 (1.3)	19.6 (1.2)	19.6 (1.2)	0.13	19.5 (1.5)	19.6 (1.3)	19.6 (1.2)	19.6 (1.2)	19.4 (1.5)	19.5 (1.4)	19.6 (1.1)	19.5 (1.2)	0.45	0.27
*Physical function*															
Physical fitness, *n*	*452*	*492*	*487*	*447*		*276*	*297*	*294*	*264*	*176*	*195*	*193*	*183*		
Physical fitness, IFIS, very poor/poor	128 (28%)	85 (17%)*	87 (18%)^#^	81 (18%)	** < 0.001**	70 (25%)	52 (33%)	47 (16%)	50 (19%)	58 (33%)	33 (17%)	39 (20%)	31 (17%)	0.65	0.09
Physical fitness, IFIS, Average/good/very good	324 (72%)	407 (83%)	401 (82%)	366 (82%)		206 (75%)	245 (83%)	247 (84%)	214 (81%)	118 (67%)	162 (83%)	154 (80%)	152 (83%)		
IPAQ-SF, *n*	*356*	*371*	*350*	*340*		*216*	*224*	*214*	*202*	*140*	*147*	*136*	*138*		
Physical activity, IPAQ-SF, MET min/wk	4243.4 (6853.9)	4758.8 (7391.9)	4103.9 (6130.2)	3804.2 (5610.0)	0.28	4439.0 (7389.7)	4580.8 (7281.1)	3906.2 (6034.5)	4059.9 (6136.2)	3941 (5946.1)	5030.0 (7574.7)	4414.9 (6287.6)	3429.9 (4732.5)	0.88	0.50
*Participation, n*	*440*	*485*	*477*	*436*		*269*	*292*	*287*	*259*	*171*	*193*	*190*	*177*		
USER-P, Frequency	28.0 (11.1)	29.3 (11.1)	29.5 (10.8)^#^	30.3 (10.8)	** < 0.001**	27.6 (11.2)	29.8 (11.1)	29.4 (10.4)	30.1 (10.8)	28.7 (11.0)	28.5 (11.2)	29.8 (11.3)	30.6 (10.8)	0.74	0.04
USER-P, Restrictions	79.4 (21.0)	85.3 (17.9)	86.1 (18.9)*^#^	86.6 (18.0)^†^	** < 0.001**	80.9 (20.2)	86.1 (18.3)	86.9 (18.1)	86.9 (18.4)	77.1 (22.1)	84.1 (17.4)	84.9 (19.9)	86.2 (17.4)	0.20	0.16
USER-P, Satisfaction	64.6 (19.5)	68.2 (18.6)*	68.4 (19.1)^#^	70.0 (18.8)	** < 0.001**	63.8 (19.2)	68.3 (19.2)	68.2 (18.3)	69.4 (19.0)	65.8 (19.9)	68.2 (17.7)	68.6 (20.4)	70.7 (18.6)	0.38	0.54
*Employment status, n*	*345*	*320*	*299*	*216*		*190*	*183*	*176*	*124*	*155*	*137*	*123*	*92*		
IPCQ, not or partially returned to work	244 (71%)	158 (49%)	93 (31%)	65 (30%)	** < 0.001**	107 (56%)	64 (35%)	47 (27%)	32 (26%)	137 (88%)	94 (69%)	46 (37%)	33 (36%)	** < 0.001**	** < 0.001**
iPCQ, fully returned to work	101 (29%)	162 (51%)	206 (69%)	151 (70%)		83 (44%)	119 (65%)	129 (73%)	92 (74%)	18 (12%)	43 (31%)	77 (63%)	59 (64%)		
*HRQoL*															
*EQ-5D-5L, n*	*442*	*482*	*479*	*437*		*274*	*288*	*289*	*260*	*168*	*194*	*190*	*177*		
Index value	0.75 (0.24)	0.79 (0.21)*	0.80 (0.21)*^#^	0.80 (0.22)	** < 0.001**	0.76 (0.23)	0.80 (0.21)	0.81 (0.20)	0.80 (0.21)	0.72 (0.26)	0.77 (0.22)	0.79 (0.23)	0.79 (0.23)	0.25	0.09
EQ-VAS	68.5 (19.5)	73.5 (17.9)*	74.0 (17.5)^#^	73.4 (18.2)	** < 0.001**	69.1 (18.9)	73.6 (17.9)	74.3 (16.8)	73.4 (18.4)	67.6 (20.6)	73.3 (17.9)	73.6 (18.5)	73.5 (17.9)	0.74	0.35
*SF-36, n*	*434*	*481*	*476*	*441*		*274*	*292*	*286*	*262*	*161*	*190*	*191*	*179*		
Physical component summary	41.3 (10.5)	43.4 (10.5)*	44.8 (10.4)*^#^	44.7 (10.6)^†^	** < 0.001**	42.1 (10.5)	44.2 (10.3)	45.4 (10.4)	45.4 (10.9)	40.1 (10.4)	42.1 (10.6)	44.0 (10.5)	43.7 (10.1)	0.10	0.23
Mental component summary	46.8 (11.9)	48.3 (11.3)*	48.6 (11.1)^#^	49.2 (10.1)	** < 0.001**	46.0 (11.7)	47.2 (11.2)	48.5 (10.5)	48.9 (9.5)	48.0 (12.0)	50.0 (11.3)	48.8 (11.9)	49.6 (10.9)	0.21	0.10

The data comprise raw test outcomes and are presented as mean (standard deviation) or n (%). Physical activity from the IPAQ-SF was expressed as MET-minutes/week using the formula: (3.3*walking minutes*walking) + (4.0*moderate-intensity activity minutes*moderate days) + (8.0*vigorous-intensity activity minutes*vigorous-intensity days). Employment status is presented for patients with a paid job pre-COVID-19. Categorical outcomes on the mMRC dyspnea scale, IFIS, and recovery status, and the domain scores in EQ-5D-5L and SF-36 questionnaires are presented in supplementary table S3A-S3B. *P* values are obtained from Generalized Estimating Equations analysis, a *P* value less than 0.00417 was considered statistically significant and is indicated in bold. *indicates a significant difference as compared to the previous study visit, ^#^ indicates a significant difference between the 3-month and 1-year study visits, and ^†^between the 6-month and 2-year study visits

*PROMs* Patient-Reported Outcome Measures, *ICU* Intensive Care Unit, *FAS* Fatigue Assessment Scale, *mMRC* Modified Medical Research Council dyspnea scale, *HADS-A* Hospital Anxiety and Depression Scale-subscale Anxiety, *HADS-D* Hospital Anxiety and Depression Scale-subscale Depression, *IES-R* Impact of Event Scale-Revised, *PTSD* Posttraumatic Stress Disorder, *CFQ* Cognitive Failures Questionnaire, *PSQI* Pittsburgh Sleep Quality Index, *BI* Barthel Index, *IFIS* International Fitness Scale, *IPAQ-SF* International Physical Activity Questionnaire-Short Form, *MET* Metabolic Equivalent of Task, *USER-P* Utrecht Scale for Evaluation of Rehabilitation-Participation, *iPCQ* iMTA Productivity Cost Questionnaire, *HRQoL* Health-Related Quality of Life, *EQ-5D-5L* 5-level EuroQoL-5D questionnaire, *EQ-VAS* EQ-Visual Analogue Scale, *SF-36* 36-item Short Form Health Survey

#### ICU- vs. non-ICU-treated patients

**Fig. 2:**
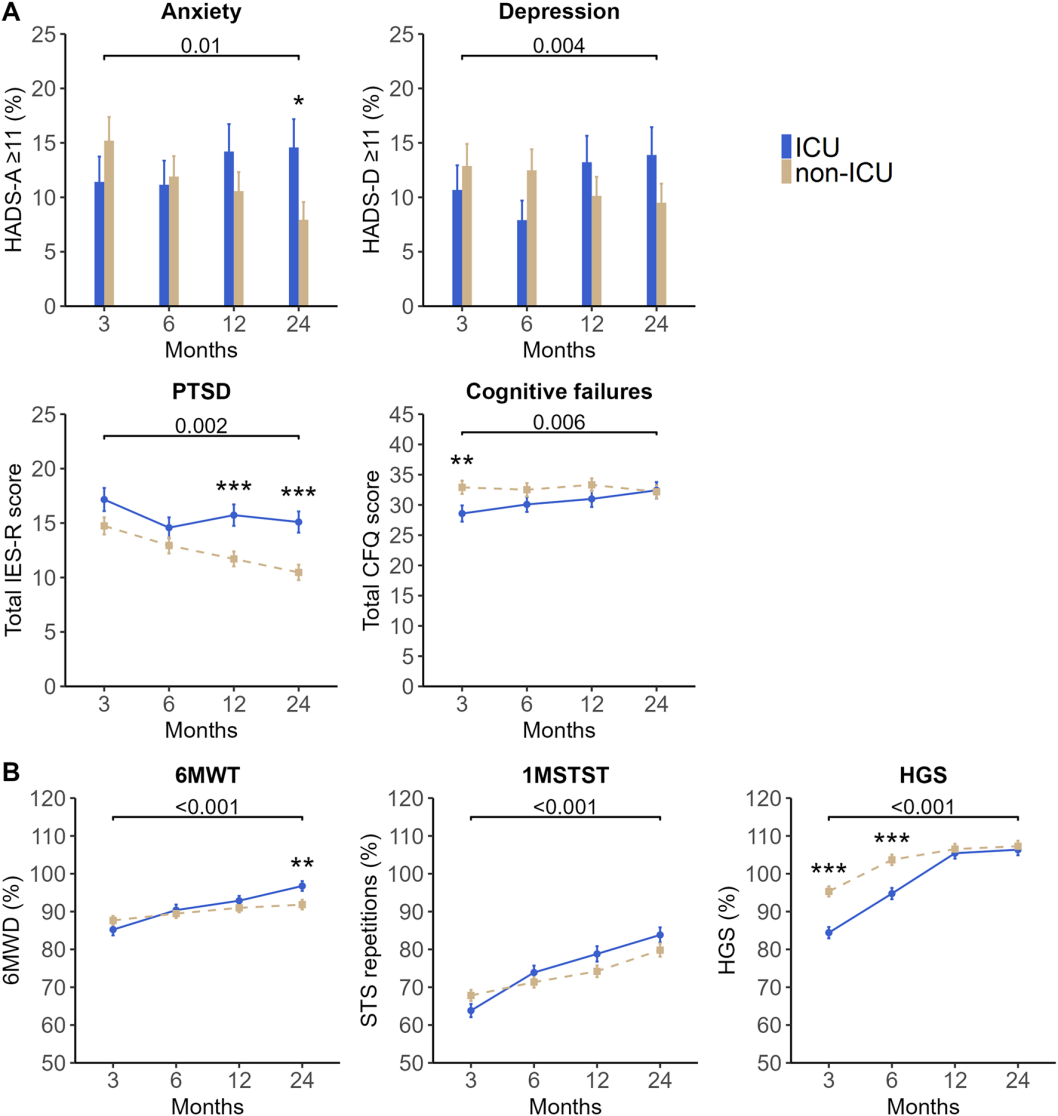
Trajectories of A: mental health and cognition and B: physical function in ICU- and non-ICU-treated patients for COVID-19 up to 2 years after hospital discharge. Data are presented as estimated proportions or estimated means with standard errors obtained from Generalized Estimating Equations analysis. **A** Estimated proportions (patients with HADS-A ≥ 11 and HADS-D ≥ 11) and estimated means (total IES-R score and total CFQ score) are adjusted for age and sex, the fixed value for age was 60 years. **B** Data are presented as the percentage of normative values reached in 6MWT, 1MSTST, and HGS. Normative values in 6MWT are calculated using sex-, age-, height-, and weight-stratified equations described by Enright and Sherill [44], in 1MSTST using sex- and age-stratified reference values described by Strassman and colleagues [45], and in HGS using sex- and age-stratified reference values described by Dodds and colleagues [46]. We compared the 2-year trajectories between the ICU and non-ICU groups and the *p* value is presented above the horizontal brackets in each panel. A significant group difference at each time point is indicated by * < 0.05, ** < 0.01, and *** < 0.001. Within group trajectories are further presented in Supplementary Table S4. *ICU* Intensive Care Unit, *HADS-A* Hospital Anxiety and Depression Scale-subscale Anxiety, *HADS-D* Hospital Anxiety and Depression Scale-subscale Depression, *IES-R* Impact of Event Scale-Revised, *CFQ* Cognitive Failures Questionnaire, *6MWT* 6 Min Walk Test, *6MWD* 6 Min Walk Distance, *1MSTST* 1 Min Sit-To-Stand Test, *STS* Sit-To-Stand, *HGS* Handgrip Strength

On average, the proportion of patients who had not yet fully returned to work was significantly higher in the ICU group than in the non-ICU group (*p* < 0.001); other outcomes were comparable (Table 3). Over time, as for mental health, Fig. 2A presents the group trajectories of PTSD and cognitive failures scores and the proportion of patients with depression and anxiety (Supplementary Table S4); after Bonferroni correction, only PTSD recovery was significantly slower in the ICU than in the non-ICU group. Moreover, the ICU group was less likely to fully return to work over time compared to the non-ICU group (OR 0.26 [95%CI 0.13–0.51], *p* < 0.001). At 2 years, outcomes did not differ significantly between groups.

### Objective study measurements

#### Total cohort

Cognitive and physical function, except for the DEMMI, outcomes improved significantly over time in the total cohort (Supplementary Table S5). At 2 years, 12% (57/464) of patients had cognitive deficits and patients reached 95% of norm in 6MWD, 83% in 1MSTST, and 108% in HGS, and the mean DEMMI score was 89/100.

#### ICU- vs. non-ICU-treated patients

On average, the ICU group had a significantly higher proportion of patients with desaturation ≥ 4% during the 6MWT (*p* < 0.001) and a lower mean percentage of norm HGS (*p* = 0.002) than the non-ICU group Supplementary Table S5). Over time, the ICU group showed significantly more improvement in the percentages of norm reached in the 6MWT (estimated mean difference 7.7% [95%CI 4.8–10.7], *p* < 0.001), 1MSTST (8.0% [3.7–12.3], *p* < 0.001), and HGS (10.0% [6.3–13.7], *p* < 0.001) compared to the non-ICU group (Fig. 2B); trajectories of cognitive function and DEMMI scores were comparable (Supplementary Table S5). At 2 years, the ICU group reached significantly higher levels in the percentage of norm 6MWD (estimated mean 96.7% [1.3] vs. 91.4% [1.3], *p* = 0.003) than the non-ICU group, but not in other cognitive and physical outcomes.

### Routine follow-up data

The PFT parameters and radiographic abnormalities for the total cohort at each visit are shown in Supplementary Table S6. Patients without signs of residual radiological or pulmonary function abnormalities were discharged from regular follow-up. Fifty-five patients with poor initial pulmonary recovery underwent repeated PFT and radiographic imaging up to 2-year follow-up, showing significant continuous improvement in PFT parameters and radiographic abnormalities; however, the latter was not significant after Bonferroni correction (Supplementary Table S7).

### Risk factors for long-term health problems after COVID-19

**Fig. 3:**
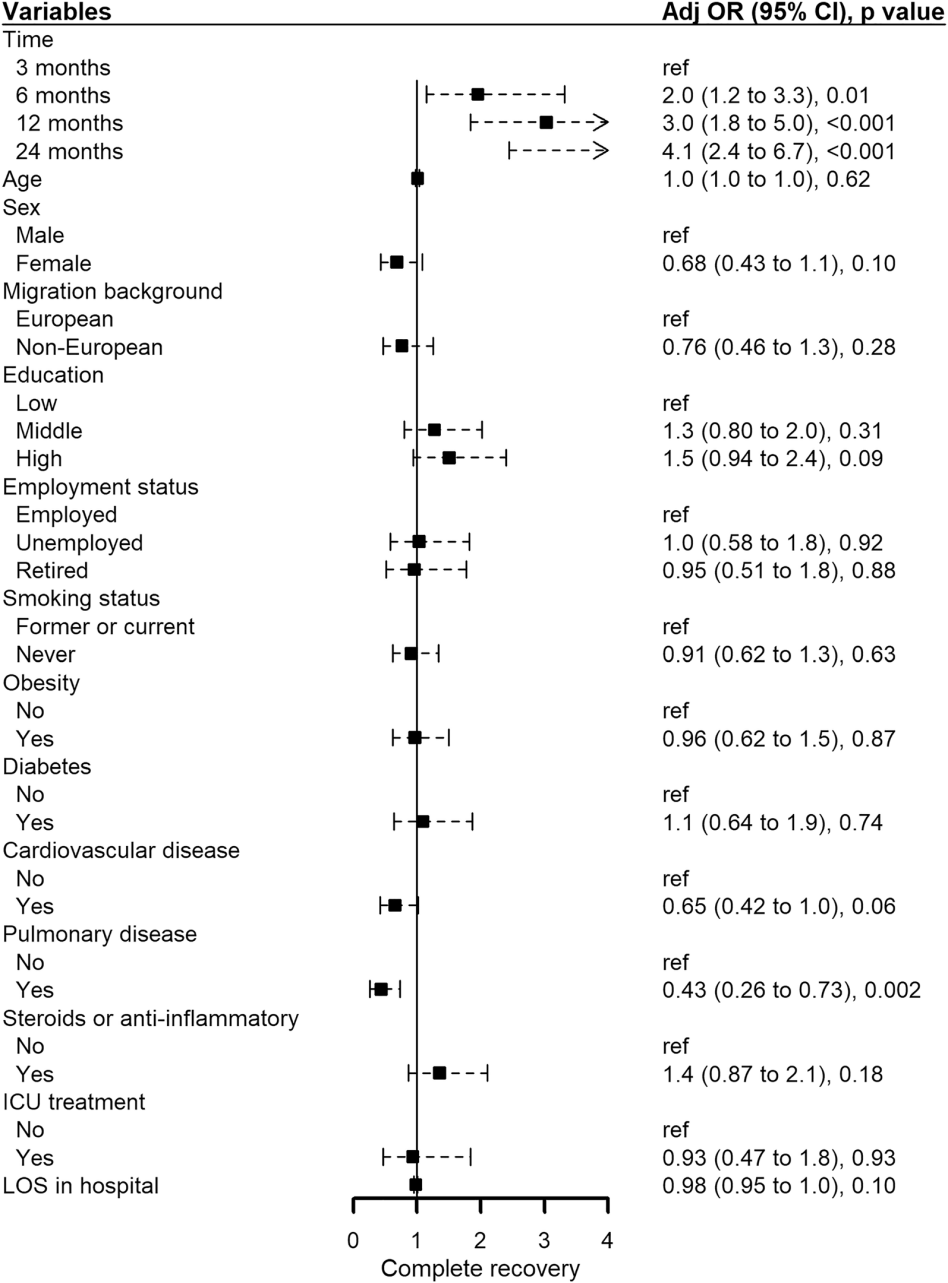
Forest plot presenting risk factors for self-reported recovery status from COVID-19. Data are obtained using multivariable Generalized Estimating Equations analysis. Recovery status from COVID-19 was assessed with the Core Outcome Measure for Recovery [21]. Recovery was dichotomized into complete recovered and not complete recovered (not recovered at all, somewhat recovered, half recovered, or mostly recovered). *Adj OR* adjusted odds ratio, *CI* confidence interval, *ICU* Intensive Care Unit, *LOS* length of stay (in days)

Over time (overall *p* < 0.001), the percentage of patients reporting complete recovery from COVID-19 increased; patients with pre-existing pulmonary disease were less likely to recover completely (OR 0.43 [95%CI 0.26–0.73], *p* = 0.002) (Fig. 3). No other factors were associated with complete recovery; recovery status did not differ between ICU- and non-ICU-treated patients. Forest plots presenting risk factors for fatigue, cognitive failures, sleep quality, and HRQoL are shown in Supplementary Figure S2. Female sex (beta 3.0 [95%CI 1.4–4.6], *p* < 0.001), pre-existing cardiovascular disease (1.9 [0.50–3.4], *p* = 0.008), and pulmonary disease (3.7 [2.1–5.3], *p* < 0.001) were associated with more fatigue; longer follow-up time (overall *p* < 0.001) and older age (− 0.10 [− 0.18 to − 0.01], *p* = 0.03) with less fatigue (Figure S2A). Female sex (7.5 [4.1–11.0], *p* < 0.001) and pre-existing pulmonary disease (7.6 [4.3–10.9], *p* < 0.001) were associated with more cognitive failures, older age (− 0.22 [− 0.39 to − 0.05], *p* = 0.01) and pre-existing obesity (− 3.1 [− 6.1 to − 0.002], *p* = 0.05) with less cognitive failures (Figure S2B). Female sex (1.8 [1.1–2.5], *p* < 0.001), non-European background (1.1 [0.3–1.9], *p* = 0.008), and pre-existing pulmonary disease (1.3 [0.6–2.0], *p* < 0.001) were associated with poorer sleep quality, longer follow-up time with better sleep quality (overall *p* = 0.01) (Figure S2C). Female sex (− 0.04 [− 0.08 to − 0.002], *p* = 0.04), non-European background (− 0.05 [− 0.09 to − 0.002], *p* = 0.04), being unemployed (vs employed, − 0.07 [− 0.12 to − 0.02], *p* = 0.009), pre-existing cardiovascular disease (− 0.04 [− 0.08 to − 0.01], *p* = 0.02), pre-existing pulmonary disease (− 0.11 [− 0.15 to − 0.06], *p* < 0.001), and a longer hospital stay (− 0.001 [− 0.002 to < − 0.001], *p* = 0.05) were associated with poorer HRQoL, and a longer follow-up time (overall *p* < 0.001) with better HRQoL (Figure S2D).

## Discussion

In this multicenter cohort study we comprehensively evaluated long-term health outcomes in 650 patients hospitalized for COVID-19 up to 2 years post-discharge, including a comparison between ICU- and non-ICU-treated patients. Many health outcomes improved over time. Nonetheless, 73% of the patients had not completely recovered from COVID-19 at 2 years. Despite good physical recovery in most patients, long-term neurocognitive complaints, dyspnea, fatigue, and poor sleep quality persisted in many. ICU-treated patients tended to show slower recovery of neurocognitive symptoms, mental health outcomes, and resumption of work compared to non-ICU-treated patients, while showing more improvements in physical outcomes. Yet, overall, outcomes were comparable between groups at 2-year follow-up. Particularly female sex and pre-existing pulmonary disease were risk factors for poorer health outcomes.

In line with our previous findings [50], we found that ICU-treated patients showed more improvements in physical tests than non-ICU-treated patients. ICU-treated patients had the poorest post-discharge outcomes, with a higher potential for improvement. Moreover, they generally had good prior performance status, allowing them to survive ICU treatment. Last, most ICU-treated patients received intensive rehabilitation [50], resulting in reaching (near) normative levels over time, comparable to the total cohort, which may suggest adequate physical rehabilitation.

As for mental health, ICU-treated patients showed slower recovery in PTSD and there was a tendency toward increasing proportions of anxiety and depression over time compared with non-ICU-treated patients, in line with our previous findings [51] and those of another COVID-19 post-ICU cohort describing deteriorating mental health outcomes from 1 to 2 years of follow-up [52]. Thus, ICU-treated patients may require extended monitoring for long-term mental health issues beyond 2 years potentiating timely interventions.

Regarding neurocognitive problems, the proportion of patients with cognitive failures and symptoms of memory or concentration problems was comparable between groups at 2 years, being prevalent in our entire study group. However, cognitive failures tended to increase over time in ICU-treated patients, as did self-reported memory and concentration problems. Moreover, ICU-treated patients had significantly more difficulties resuming work, building on previous findings [53], potentially related to this higher neurocognitive symptom burden [54].

Our findings may suggest unmet needs regarding neurocognitive rehabilitation, emphasizing the need for further development of COVID-19 aftercare strategies. Notably, in the Netherlands, COVID-19 care pathways primarily anticipated physical problems, in contrast to mental and cognitive problems. As for future pandemics, proactive strategies using a comprehensive assessment of physical, mental, and cognitive functioning should be considered in aftercare strategies.

ICU treatment was not an independent risk factor for the selected long-term health problems in our study. In contrast, several studies have shown that more severe acute COVID-19 is associated with a higher risk for health problems beyond 1 year [4, 17]. This discrepancy may be attributed to heterogeneity in study populations, methodologies, and measurements. The increased rate of persistent complaints in ICU-treated patients is frequently attributed to the superimposed effects of the PICS. However, similar long-term health problems are also experienced by patients with a mild SARS-CoV-2 infection, who do not require ICU admission or hospitalization [55]. Therefore, it seems less plausible to attribute these long-term health problems to PICS [56].

The most important determinants for long-term health problems were female sex and pre-existing pulmonary disease. We consistently [4, 16] identified female sex as major risk factor, except for self-reported complete recovery. Contrary, the PHOSP-COVID study did find a negative association between female sex and complete recovery 1 year after hospitalization [2]. This difference may resolve beyond 1 year or be due to using a different recovery scale. As for underlying pulmonary disease, some studies showed that particularly patients with asthma are at risk for poorer health outcomes after COVID-19 [16]; unfortunately, our data did not allow differentiation of pulmonary diseases to assess this into more depth.

Last, we found non-European migration background to be associated with poorer sleep quality and HRQoL, but not with other health outcomes. A few studies on health problems after COVID-19 suggest that ethnic minorities are disproportionately impacted, but data from European countries are scarce [57]. As we do not unequivocally find a relation between migration background and the assessed health outcomes, it remains unclear whether the found associations were COVID-19 specific, or attributable to pre-existing social and health inequalities, and thus requires further study.

Overall, the vast majority of our patients (88%) reported at least one new or worsened COVID-19-related symptom 2 years post-discharge, compared to 55% to 84% in other reports [4, 13]. Consistently, impaired fitness, neurocognitive problems, fatigue, dyspnea, poor sleep quality, and reduced HRQoL were identified as most prominent health problems 2 years after hospitalization for COVID-19 [4, 13, 58].

Noteworthy, we observed some discrepancies between self-reported symptoms and objectively assessed outcomes, such as between dyspnea and pulmonary function, self-reported muscle weakness and HGS, and self-reported impaired fitness and objectively assessed aerobic capacity. Factors contributing to this disparity include individual interpretations and experiences of symptoms as well as insufficient understanding of the underlying biological etiology of persistent health problems after COVID-19. Self-reported measures might capture a broader range of sensations, whereas objective tests often focus on specific aspects of functioning. Nonetheless, the subjective experience of health problems is essential as it reflects the extent to which they hinder daily functioning and highlights the need for a better understanding of the etiology of the persistent problems [59].

Strengths of this study include its prospective multicenter design with 2-year follow-up of a large cohort of ICU- and non-ICU-treated patients, the comprehensive evaluation of both PROMs and objective measures, and high response rate (78% [509/650]) up to 2 years. We were able to perform multivariable analyses to identify risk factors for prominent health problems. Study limitations include the absence of control groups of individuals without COVID-19 and non-hospitalized individuals with COVID-19 and the inability to compare our outcomes with pre-COVID-19 levels, only to the first assessment and reference values. Since most patients were unvaccinated against COVID-19 prior to hospital admission, our findings might be less generalizable to those who had been vaccinated beforehand, as vaccination appears to reduce the risk of long-term health problems [60]. Selection bias might play a role in our study as we included a higher percentage of ICU patients (42%), due to high inclusion rate from an academic hospital, compared to the average ICU admissions across all Dutch hospitals (14%) which limits the representativeness of our cohort and might overestimate poor outcomes. However, this allowed for comparison between ICU- and non-ICU-treated patients on long-term health outcomes. We observed no noticeable disparity on health outcomes at 2 years between these groups; therefore, overestimation of poor outcomes is unlikely to play a major role. In addition, we lack data on the eligible recruitment population due to the surge of patients admitted to the participating centers. However, recruitment of study participants occurred independently of the patient’s recovery status and primarily depended on availability of research personnel. Moreover, our participant characteristics align with those of the average Dutch patients hospitalized for COVID-19 [18]. Also, as one of the inclusion criteria was sufficient knowledge of the Dutch or English language, ethnic minorities are somewhat underrepresented in our study compared to the demographics of the recruitment area. Nonetheless, the ethnic minority group still comprised 29% of the participants allowing for assessment of differences between ethnicity groups. Furthermore, severity of symptoms was only assessed at the 2-year follow-up, after we concluded that given the high prevalence of persisting symptoms, a more detailed longitudinal assessment would have been beneficial.

In conclusion, most health outcomes improved over the 2 years after hospitalization for COVID-19. Nonetheless, many patients suffer from long-term health problems, with neurocognitive symptoms, dyspnea, fatigue, and poor sleep quality among the most frequent problems at 2 years and a significant proportion of patients still report incomplete recovery. Despite slower recovery in some outcomes, most 2-year health outcomes were comparable between ICU- and non-ICU-treated patients. Generally, while physical rehabilitation seems adequate, there is a need for targeted aftercare strategies addressing a variety of long-term problems and continuous research into effective treatments, including more tailored rehabilitative support and pharmacological treatment options. Moreover, our study underlines the importance of prolonged follow-up to monitor recovery from COVID-19 beyond 2 years. Therefore, we extended our study with yearly follow-up, addressing in particular the main persisting health problems.

## Supplementary Information

Additional file 1: Figure S1. Flowchart of COVID-19 patients that received post-discharge follow-up in the hospital. The first follow-up visit was generally scheduled around 6 weeks post-discharge. For patients with persistent residual pulmonary abnormalities, follow-up was continued around 3 months, 6 months, 1 year, and 2 years after hospital discharge. After each visit, patients with no or minimal residual pulmonary abnormalities were discharged from further follow-up. Pulmonary function test (PFT) comprised the assessment of spirometry and/or gas exchange. Figure S2. Forest plots presenting risk factors of A: fatigue, B: cognitive failures, C: sleep quality, and D: EQ-5D index value. Data are obtained using multivariable Generalized Estimating Equations analysis. Fatigue was assessed with the Fatigue Assessment Scale, the total FAS score ranges from 0 to 50 with higher scores representing more symptoms of fatigue. Cognitive failures were assessed with the Cognitive Failures Questionnaire, the total CFQ score ranges from 0 to 100 with higher scores representing more cognitive failures. Sleep quality was assessed with the Pittsburgh Sleep Quality Index, the total PSQI score ranges from 0 to 21 with higher scores representing poorer sleep quality. Health-related quality of life was assessed with the 5-level EuroQoL-5D questionnaire, a EQ-5D index value of 0 indicates death and 1 perfect health; negative scores indicate a health status worse than death. Adj β, Adjusted Beta; CI, Confidence Interval; ICU, Intensive Care Unit; LOS, Length Of Stay. Table S1. Trajectories of self-reported recovery and symptoms in ICU- and non-ICU-treated patients for COVID-19 up to 2 years after hospital discharge. Table S2. The severity of symptoms in COVID-19 patients at 2 years after hospital discharge. Table S3A. Categorical outcomes on the mMRC dyspnea scale, IFIS, and recovery status questionnaires in patients with COVID-19 up to 2 years after hospital discharge. Table S3B. Domain scores of the EQ-5D-5L and SF-36 questionnaires in patients with COVID-19 up to 2 years after hospital discharge. Table S4. Trajectories of mental health and physical function up to 2 years after hospitalization within ICU- and non-ICU-treated COVID-19 patients. Table S5. Outcomes of objectively assessed cognitive and physical function in COVID-19 patients up to 2 years after hospital discharge. Table S6. Pulmonary function testing and radiologic outcomes in the total cohort up to 2 years after hospitalization for COVID-19. Table S7. Pulmonary function testing and radiological outcomes in 55 patients with initial poor pulmonary recovery who continued follow-up up to 2 years after hospitalization for COVID-19

## Data Availability

The datasets used and/or analyzed during the current study are available from the corresponding author on reasonable request.

## Abbreviations

COVID-19: Coronavirus Disease 2019
ICU: Intensive Care Unit
CO-FLOW: COvid-19 Follow-up care paths and Long-term Outcomes Within the Dutch healthcare system
PROMs: Patient-Reported Outcome Measures
GEE: Generalized Estimating Equations
SD: Standard Deviation
IQR: Interquartile Ranges
Adj OR: Adjusted Odds Ratio
CI: Confidence Intervals
LOS: Length Of Stay
PICS: Post-Intensive Care Syndrome
NA: Not Applicable
6MWT: 6 Min Walk Test
6MWD: 6 Min Walk Distance
1MSTST: 1 Min Sit-To-Stand Test
STS: Sit-To-Stand
HGS: Handgrip Strength
FAS: Fatigue Assessment Scale
mMRC: Modified Medical Research Council dyspnea scale
HADS-A: Hospital Anxiety and Depression Scale-subscale Anxiety
HADS-D: Hospital Anxiety and Depression Scale-subscale Depression
IES-R: Impact of Event Scale-Revised
PTSD: Posttraumatic Stress Disorder
CFQ: Cognitive Failures Questionnaire
PSQI: Pittsburgh Sleep Quality Index
BI: Barthel Index
IFIS: International Fitness Scale
IPAQ-SF: International Physical Activity Questionnaire-Short Form
MET: Metabolic Equivalent of Task
USER-P: Utrecht Scale for Evaluation of Rehabilitation-Participation
iPCQ: IMTA Productivity Cost Questionnaire
HRQoL: Health-Related Quality of Life
EQ-5D-5L: 5-Level EuroQoL-5D questionnaires
EQ-VAS: EQ-Visual Analogue Scale
SF-36: 36-Item Short Form Health Survey
FVC: Forced Vital Capacity
LLN: Lower Limit Of Normal
FEV_1_: Forced Expiratory Volume in 1 s
DLCOc: Diffusing Capacity of the Lung for Carbon Monoxide adjusted for hemoglobin
GGO: Ground-Glass Opacity
