# Supplementary material for: Long-term health outcomes of COVID-19 in ICU- and non-ICU-treated patients up to 2 years after hospitalization: a longitudinal cohort study (CO-FLOW)
Source: J Intensive Care. 2024 Nov 8;12:47. doi: 10.1186/s40560-024-00748-w (PMC11546104; doi:10.1186/s40560-024-00748-w)
Supplement: Supplementary file 1 — Additional file 1: Figure S1. Flowchart of COVID-19 patients that received post-discharge follow-up in the hospital. The first follow-up visit was generally scheduled around 6 weeks post-discharge. For patients with persistent residual pulmonary abnormalities, follow-up was continued around 3 months, 6 months, 1 year, and 2 years after hospital discharge. After each visit, patients with no or minimal residual pulmonary abnormalities were discharged from further follow-up. Pulmonary function test (PFT) comprised the assessment of spirometry and/or gas exchange. Figure S2. Forest plots presenting risk factors of A: fatigue, B: cognitive failures, C: sleep quality, and D: EQ-5D index value. Data are obtained using multivariable Generalized Estimating Equations analysis. Fatigue was assessed with the Fatigue Assessment Scale, the total FAS score ranges from 0 to 50 with higher scores representing more symptoms of fatigue. Cognitive failures were assessed with the Cognitive Failures Questionnaire, the total CFQ score ranges from 0 to 100 with higher scores representing more cognitive failures. Sleep quality was assessed with the Pittsburgh Sleep Quality Index, the total PSQI score ranges from 0 to 21 with higher scores representing poorer sleep quality. Health-related quality of life was assessed with the 5-level EuroQoL-5D questionnaire, a EQ-5D index value of 0 indicates death and 1 perfect health; negative scores indicate a health status worse than death. Adj β, Adjusted Beta; CI, Confidence Interval; ICU, Intensive Care Unit; LOS, Length Of Stay. Table S1. Trajectories of self-reported recovery and symptoms in ICU- and non-ICU-treated patients for COVID-19 up to 2 years after hospital discharge. Table S2. The severity of symptoms in COVID-19 patients at 2 years after hospital discharge. Table S3A. Categorical outcomes on the mMRC dyspnea scale, IFIS, and recovery status questionnaires in patients with COVID-19 up to 2 years after hospital discharge. Table S3B. Domain [file 40560_2024_748_MOESM1_ESM.docx]

**Supplementary materials**

**Long-term health outcomes of COVID-19 in ICU- and non-ICU-treated patients up to 2 years after hospitalization: a longitudinal cohort study (CO-FLOW)**

J.C. Berentschot^1*^, L.M. Bek^2*^, M.H. Heijenbrok-Kal^2,3^, J. van Bommel^4^, G.M. Ribbers^2,3^, J.G.J.V. Aerts^1^, M.E. Hellemons^1**^, H.J.G. van den Berg-Emons^2**^ on behalf of the CO-FLOW collaboration Group

^*^ Authors share first authorship; ^**^ Authors share senior authorship

**Affiliations:**

^1^Department of Respiratory Medicine, Erasmus Medical Center, Rotterdam, The Netherlands.

^2^Department of Rehabilitation Medicine, Erasmus Medical Center, Rotterdam, The Netherlands.

^3^Rijndam Rehabilitation, Rotterdam, The Netherlands.

^4^Department of Intensive Care, Erasmus Medical Center, Rotterdam, The Netherlands.

**Contents**

**Figure S1. Flowchart of COVID-19 patients that received post-discharge follow-up in the hospital.** **................... P2**

**Figure S2.** **Forest plots presenting risk factors of A: fatigue, B: cognitive failures, C: sleep quality, and D: EQ-5D index value. ...................................................................................................................................................... P3**

**Table S1.** **Trajectories of self-reported recovery and symptoms in ICU- and non-ICU-treated patients for COVID-19 up to 2 years after hospital discharge. .......................................................................................................... P5**

**Table S2. The severity of symptoms in COVID-19 patients at 2 years after hospital discharge. ........................... P7**

**Table S3A. Categorical outcomes on the mMRC dyspnea scale, IFIS, and recovery status questionnaires in COVID-19 patients up to 2 years after hospital discharge. ............................................................................................ P9**

**Table S3B. Domain scores of the EQ-5D-5L and SF-36 questionnaires in COVID-19 patients up to 2 years after hospital discharge. .......................................................................................................................................... P10**

**Table S4. Trajectories of mental health and physical function up to 2 years after hospitalization within ICU- and non-ICU-treated COVID-19 patients. ............................................................................................................... P11**

**Table S5. Outcomes of objectively assessed cognitive and physical function in COVID-19 patients up to 2 years after hospital discharge. .................................................................................................................................. P13**

**Table S6. Pulmonary function testing and radiologic outcomes in the total cohort up to 2 years after hospitalization for COVID-19. .......................................................................................................................... P14**

**Table S7. Pulmonary function testing and radiological outcomes in 55 patients with initial poor pulmonary recovery who continued follow-up up to 2 years after hospitalization for COVID-19. ...................................... P15**

**CO-FLOW Collaboration Group. ....................................................................................................................... P16**


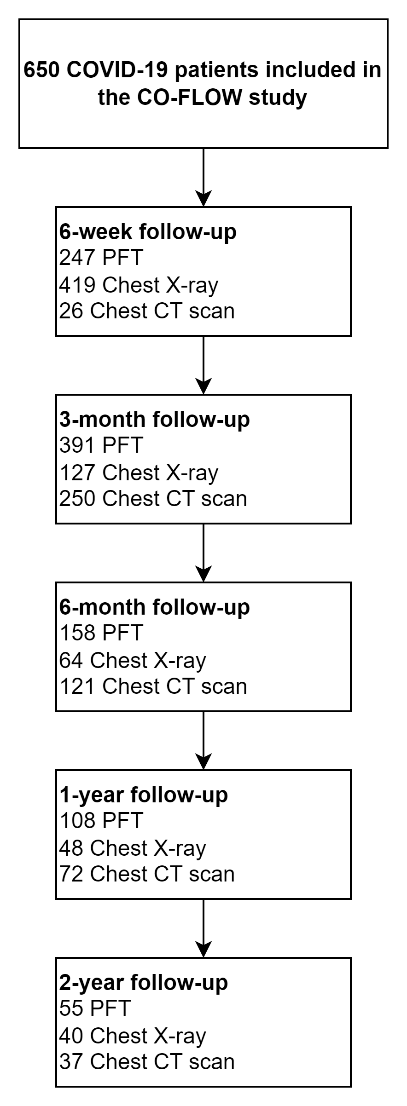


**Figure S1.** Flowchart of COVID-19 patients that received post-discharge follow-up in the hospital. The first follow-up visit was generally scheduled around 6 weeks post-discharge. For patients with persistent residual pulmonary abnormalities, follow-up was continued around 3 months, 6 months, 1 year, and 2 years after hospital discharge. After each visit, patients with no or minimal residual pulmonary abnormalities were discharged from further follow-up. Pulmonary function test (PFT) comprised the assessment of spirometry and/or gas exchange

**
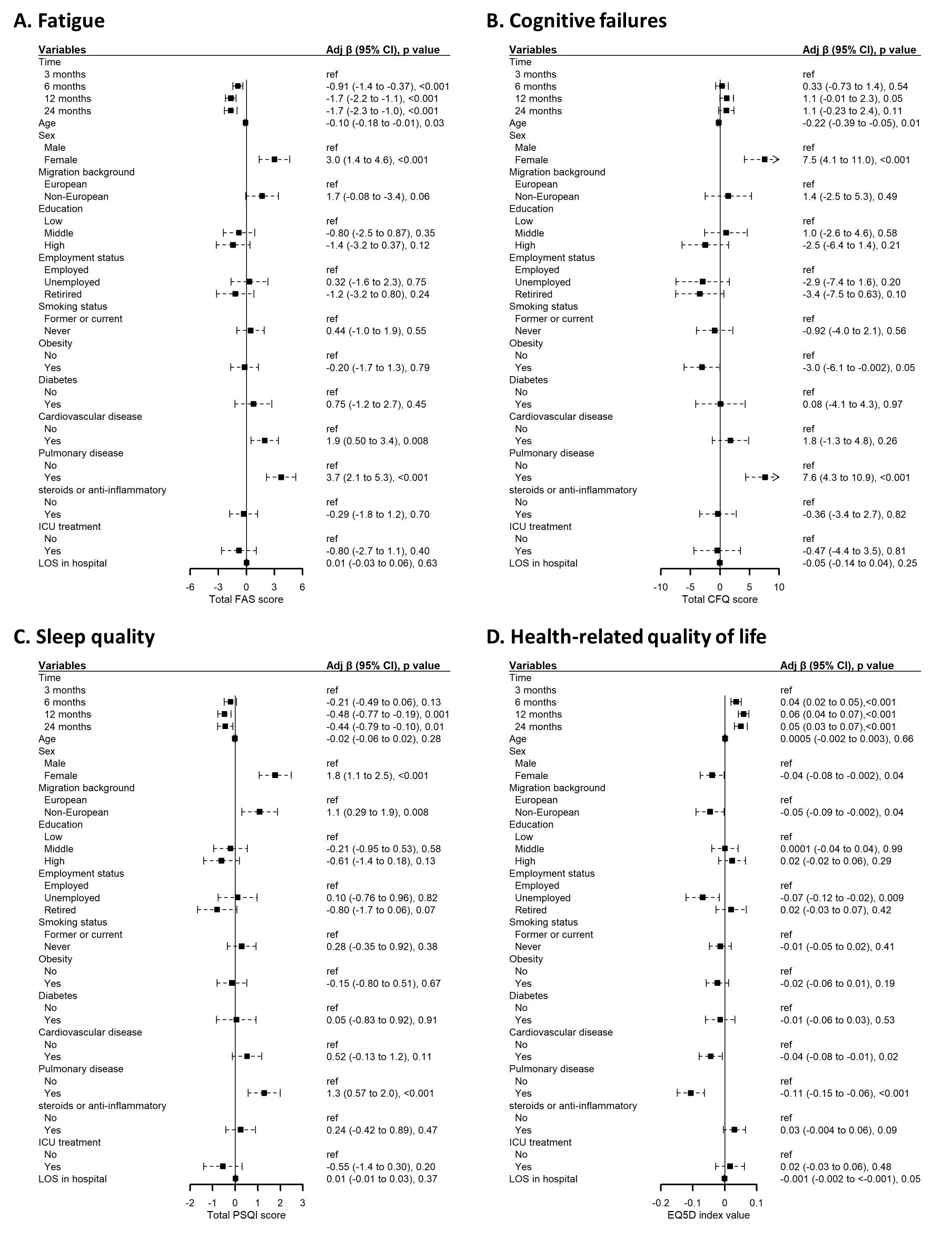
**

**Figure S2.** Forest plots presenting risk factors of A: fatigue, B: cognitive failures, C: sleep quality, and D: EQ-5D index value. Data are obtained using multivariable Generalized Estimating Equations analysis. Fatigue was assessed with the Fatigue Assessment Scale (FAS), the total FAS score ranges from 0 to 50 with higher scores representing more symptoms of fatigue. Cognitive failures were assessed with the Cognitive Failures Questionnaire (CFQ), the total CFQ score ranges from 0 to 100 with higher scores representing more cognitive failures. Sleep quality was assessed with the Pittsburgh Sleep Quality Index (PSQI), the total PSQI score ranges from 0 to 21 with higher scores representing poorer sleep quality. Health-related quality of life was assessed with the 5-level EuroQoL-5D (EQ5D) questionnaire, a EQ5D index value of 0 indicates death and 1 perfect health; negative scores indicate a health status worse than death. *Adj β* Adjusted Beta, *CI* Confidence Interval, *ICU* Intensive Care Unit, *LOS* Length Of Stay (in days)

**Table S1** Trajectories of self-reported recovery and symptoms in ICU- and non-ICU-treated patients for COVID-19 up to 2 years after hospital discharge.

|  | **Total** | | | | | **Non-ICU** | | | | **ICU** | | | | **Overall comparison**  **ICU vs**  **non-ICU** | | **Interaction ICU * Time** | |  |
| --- | --- | --- | --- | --- | --- | --- | --- | --- | --- | --- | --- | --- | --- | --- | --- | --- | --- | --- |
|  | **3**  **months** | **6**  **months** | **1**  **year** | **2**  **years** | **P value** | **3**  **months** | **6**  **months** | **1**  **year** | **2**  **years** | **3**  **months** | **6**  **months** | **1**  **year** | **2**  **years** | | **P value** | | **P value** | |
| **Recovery status, *n*** | *159* | *300* | *418* | *443* |  | *90* | *184* | *225* | *260* | *69* | *116* | *193* | *183* | |  | |  | |
| Not completely recovered | 142 (89%) | 248 (83%) | 316 (77%) | 322 (73%) | **<0.001** | 78 (87%) | 142 (77%) | 159 (74%) | 180 (69%) | 64 (93%) | 106 (91%) | 157 (81%) | 142 (78%) | | 0.003 | | 0.08 | |
| **Symptoms, *n*** | *441* | *528* | *532* | *503* |  | *275* | *311* | *310* | *300* | *166* | *218* | *222* | *203* | |  | |  | |
| ≥ 1 | 427/442 (97%) | 504/526 (96%) | 492/532 (93%) | 443/503 (88%) | **<0.001** | 264/275 (96%) | 296/308 (96%) | 281/310 (91%) | 253/300 (84%) | 163/167 (98%) | 208 (95%) | 211/222 (95%) | 190/203 (94%) | | 0.10 | | 0.08 | |
| Impaired fitness | 362 (83%) | 379 (72%)* | 346 (65%)*^#^ | 311 (62%)*^ꬹ^ | **<0.001** | 217 (79%) | 218 (70%) | 196 (63%) | 165 (58%) | 145 (88%) | 161 (74%) | 149 (67%) | 138 (68%) | | 0.02 | | 0.17 | |
| Fatigue | 116/140 (83%) | 162/241 (67%)* | 237/380 (62%)^#^ | 302/493 (61%) | **<0.001** | 74/84 (88%) | 93/140 (66%) | 146/235 (62%) | 190/292 (58%) | 42/56 (75%) | 69/101 (68%) | 91/145 (63%) | 132/201 (66%) | | 0.99 | | 0.16 | |
| Dyspnea | 87/128 (68%) | 127/237 (54%)* | 210/378 (56%) | 251/493 (51%) | **0.001** | 61/78 (78%) | 74/135 (55%) | 133/235 (56%) | 139/292 (48%) | 26/50 (52%) | 53/102 (52%) | 77/143 (54%) | 112/201 (56%) | | 0.47 | | 0.005 | |
| Muscle weakness | 253 (58%) | 258 (49%)* | 225 (42%)*^#^ | 189 (38%)^ꬹ^ | **<0.001** | 143 (52%) | 135 (43%) | 116 (38%) | 105 (35%) | 110 (66%) | 123 (57%) | 108 (49%) | 84 (41%) | | **<0.001** | | 0.23 | |
| Memory problems | 238 (54%) | 302 (57%) | 296 (56%) | 274 (55%) | 0.44 | 163 (59%) | 190 (61%) | 177 (57%) | 155 (52%) | 75 (45%) | 112 (52%) | 119 (54%) | 119 (59%) | | 0.11 | | **0.001** | |
| Concentration problems | 232 (53%) | 273 (52%) | 271 (51%) | 259 (52%) | 0.81 | 158 (58%) | 166 (54%) | 159 (51%) | 150 (50%) | 74 (45%) | 107 (49%) | 111 (50%) | 109 (54%) | | 0.19 | | 0.03 | |
| Sensory overload | 50/109 (46%) | 100/229 (44%) | 145/381 (38%) | 196/495 (40%) | 0.50 | 33/61 (54%) | 56/129 (43%) | 90/236 (38%) | 103/294 (35%) | 17/48 (35%) | 44/100 (44%) | 55/145 (38%) | 90/201 (46%) | | 0.65 | | 0.09 | |
| Joint pain | 187 (43%) | 218 (41%) | 217 (41%) | 170 (34%)^ꬹ^ | **<0.001** | 109 (40%) | 113 (36%) | 110 (36%) | 92 (31%) | 78 (48%) | 105 (48%) | 107 (48%) | 78 (38%) | | 0.002 | | 0.75 | |
| Balance problems/dizziness | 184 (42%) | 228 (44%) | 223 (42%) | 200 (40%) | 0.53 | 118 (43%) | 126 (41%) | 123 (40%) | 104 (35%) | 66 (40%) | 102 (47%) | 99 (45%) | 96 (48%) | | 0.09 | | 0.09 | |
| Sleep disturbances | 160 (36%) | 182 (35%) | 185 (35%) | 141 (28%)* | **0.002** | 108 (39%) | 119 (39%) | 101 (33%) | 73 (24%) | 52 (31%) | 63 (29%) | 83 (37%) | 68 (34%) | | 0.97 | | **<0.001** | |
| Tingling/numbness in extremities | 164 (36%) | 182 (35%) | 169 (32%) | 151 (30%) | 0.19 | 90 (32%) | 84 (27%) | 85 (27%) | 78 (26%) | 74 (43%) | 98 (45%) | 84 (38%) | 73 (37%) | | **<0.001** | | 0.23 | |
| Hair loss | 158 (36%) | 105 (20%)* | 69 (13%)*^#^ | 78 (16%) | **<0.001** | 91 (33%) | 68 (22%) | 43 (14%) | 50 (17%) | 67 (41%) | 37 (17%) | 25 (11%) | 28 (14%) | | 0.38 | | 0.04 | |
| Headache | 39/117 (33%) | 58/235 (25%) | 77/377 (20%) | 91/491 (19%) | 0.004 | 30/67 (45%) | 38/134 (28%) | 47/234 (20%) | 52/293 (18%) | 9/50 (18%) | 20/101 (20%) | 30/143 (21%) | 39/201 (20%) | | 0.11 | | 0.02 | |
| Cough | 122 (28%) | 130 (25%) | 126 (24%) | 137 (27%) | 0.32 | 73 (27%) | 81 (26%) | 69 (22%) | 79 (26%) | 49 (30%) | 49 (23%) | 57 (26%) | 58 (29%) | | 0.69 | | 0.44 | |
| Chest pain | 30/111 (27%) | 41/234 (18%) | 67/379 (18%) | 84/494 (17%) | 0.019 | 22/63 (35%) | 30/133 (23%) | 43/234 (18%) | 49/293 (17%) | 8/48 (17%) | 11/101 (11%) | 24/144 (17%) | 35/198 (17%) | | 0.09 | | 0.27 | |
| Skin rash | 113 (26%) | 145 (28%) | 154 (29%) | 128 (26%) | 0.42 | 59 (22%) | 85 (28%) | 83 (27%) | 79 (26%) | 54 (33%) | 60 (28%) | 70 (32%) | 49 (24%) | | 0.12 | | 0.01 | |
| Phlegm | 114 (26%) | 125 (24%) | 131 (25%) | 101 (20%) | 0.11 | 73 (27%) | 73 (24%) | 71 (23%) | 62 (21%) | 41 (25%) | 52 (24%) | 59 (27%) | 39 (19%) | | 0.95 | | 0.57 | |
| Vision problems | 108 (25%) | 160 (30%) | 158 (30%) | 149 (30%) | 0.05 | 73 (27%) | 98 (32%) | 90 (29%) | 85 (28%) | 35 (21%) | 62 (29%) | 67 (30%) | 64 (32%) | | 0.54 | | 0.43 | |
| Anosmia | 104 (24%) | 99 (19%) | 98 (18%) | 101 (20%) | 0.03 | 76 (28%) | 66 (21%) | 65 (21%) | 68 (23%) | 28 (17%) | 33 (15%) | 33 (15%) | 33 (16%) | | 0.01 | | 0.72 | |
| Hoarseness | 103 (23%) | 134 (25%) | 121 (23%) | 99 (20%) | 0.04 | 59 (22%) | 66 (21%) | 58 (19%) | 44 (15%) | 44 (27%) | 68 (32%) | 63 (28%) | 55 (27%)^ⴕ^ | | **<0.001** | | 0.36 | |
| Ageusia | 94 (21%) | 102 (19%) | 101 (19%) | 95 (19%) | 0.55 | 68 (25%) | 67 (22%) | 72 (23%) | 63 (21%) | 26 (16%) | 35 (16%) | 29 (13%) | 32 (16%) | | 0.009 | | 0.37 | |
| Stool problems | 78 (18%) | 96 (18%) | 84 (16%) | 80 (16%) | 0.34 | 54 (20%) | 59 (19%) | 50 (16%) | 44 (15%) | 24 (15%) | 37 (17%) | 34 (15%) | 36 (18%) | | 0.58 | | 0.44 | |
| Hearing problems | 61 (14%) | 77 (15%) | 109 (21%)*^#^ | 108 (21%) ^ꬹ^ | **<0.001** | 45 (16%) | 53 (17%) | 72 (23%) | 71 (24%) | 16 (10%) | 24 (11%) | 37 (17%) | 37 (18%) | | 0.01 | | 0.86 | |
| Anxiety/nightmares | 60 (14%) | 79 (15%) | 77 (15%) | 59 (12%) | 0.21 | 34 (12%) | 48 (15%) | 38 (12%) | 29 (10%) | 26 (16%) | 31 (14%) | 39 (18%) | 30 (15%) | | 0.24 | | 0.18 | |
| Claudication | 61 (14%) | 73 (14%) | 53 (10%) | 46 (9%) | 0.016 | 43 (16%) | 42 (14%) | 27 (9%) | 21 (7%) | 18 (11%) | 31 (14%) | 26 (12%) | 25 (12%) | | 0.34 | | 0.14 | |
| Miction problems | 40 (9%) | 66 (13%) | 65 (12%) | 64 (13%) | 0.22 | 29 (11%) | 43 (14%) | 38 (12%) | 33 (11%) | 11 (7%) | 23 (11%) | 26 (12%) | 31 (15%) | | 0.53 | | 0.17 | |

The data comprise raw test outcomes and are presented as n (%) or as n/N (%) in the case of adjusted total number. The presence of symptoms was assessed with a symptom questionnaire (Corona Symptom Checklist, CSC) on new or worsened symptoms following SARS-CoV-2 infection. The symptoms fatigue, dyspnea, headache, chest pain, and sensory overload were added to the CSC in a later stage and therefore contain lower total numbers. Recovery status from COVID-19 was dichotomized into completely recovered and not complete recovered (not recovered at all, somewhat recovered, half recovered, or mostly recovered). P values are obtained from Generalized Estimating Equations analysis, a P value less than 0.00185 was considered statistically significant and is indicated in bold. In the total cohort, ^*^ indicates a significant difference as compared to the previous study visit, ^#^ indicates a significant difference between the 3-month and 1-year study visits, and ^ꬹ^ between the 6-month and 2-year study visits. ^ⴕ^ indicates significant difference between ICU and non-ICU group at 2 years

**Table S2.** The severity of symptoms in COVID-19 patients at 2 years after hospital discharge.

|  | **Total** | | | | | **Non-ICU** | | | | | **ICU** | | | | |
| --- | --- | --- | --- | --- | --- | --- | --- | --- | --- | --- | --- | --- | --- | --- | --- |
| **Symptoms** | **N** | **Mild** | **Moderate** | **Severe** | **Very severe** | **N** | **Mild** | **Moderate** | **Severe** | **Very severe** | **N** | **Mild** | **Moderate** | **Severe** | **Very severe** |
| Impaired fitness | 254 | 61 (24%) | 108 (43%) | 67 (26%) | 18 (7%) | 140 | 34 (24%) | 57 (41%) | 41 (29%) | 8 (6%) | 114 | 27 (24%) | 51 (45%) | 26 (22%) | 10 (9%) |
| Fatigue | 253 | 40 (16%) | 105 (41%) | 85 (34%) | 23 (9%) | 143 | 22 (15%) | 60 (42%) | 49 (34%) | 12 (8%) | 110 | 18 (16%) | 45 (41%) | 36 (33%) | 11 (10%) |
| Muscle weakness | 144 | 27 (19%) | 70 (49%) | 40 (29%) | 7 (5%) | 79 | 16 (20%) | 36 (46%) | 22 (28%) | 5 (6%) | 65 | 11 (17%) | 34 (52%) | 18 (28%) | 2 (3%) |
| Memory problems | 225 | 58 (26%) | 85 (38%) | 70 (31%) | 12 (5%) | 123 | 27 (22%) | 51 (42%) | 38 (31%) | 7 (6%) | 102 | 31 (30%) | 34 (33%) | 32 (31%) | 5 (5%) |
| Concentration problems | 217 | 51 (24%) | 87 (40%) | 71 (33%) | 8 (4%) | 124 | 29 (23%) | 49 (40%) | 40 (32%) | 6 (5%) | 93 | 22 (24%) | 38 (41%) | 31 (33%) | 2 (2%) |
| Sensory overload | 170 | 41 (24%) | 66 (39%) | 52 (31%) | 11 (7%) | 92 | 22 (23%) | 33 (36%) | 33 (36%) | 4 (4%) | 78 | 19 (24%) | 33 (42%) | 19 (24%) | 7 (9%) |
| Balance problems /dizziness | 163 | 57 (35%) | 66 (41%) | 34 (21%) | 6 (4%) | 84 | 29 (35%) | 35 (42%) | 17 (20%) | 3 (4%) | 79 | 28 (35%) | 31 (39%) | 17 (22%) | 3 (4%) |
| Tingling /numbness in extremities | 120 | 30 (25%) | 57 (48%) | 23 (20%) | 10 (8%) | 64 | 18 (28%) | 31 (48%) | 11 (17%) | 4 (6%) | 56 | 12 (21%) | 26 (46%) | 12 (21%) | 6 (11%) |
| Hair loss | 61 | 22 (36%) | 21 (34%) | 13 (21%) | 5 (8%) | 40 | 14 (35%) | 12 (30%) | 12 (30%) | 2 (5%) | 21 | 8 (38%) | 9 (43%) | 1 (5%) | 3 (14%) |
| Sleep disturbances | 113 | 18 (16%) | 37 (33%) | 50 (44%) | 8 (7%) | 56 | 10 (17%) | 15 (27%) | 29 (52%) | 2 (4%) | 57 | 8 (14%) | 22 (39%) | 21 (27%) | 6 (11%) |
| Headache | 78 | 18 (23%) | 39 (50%) | 15 (19%) | 6 (8%) | 45 | 8 (18%) | 23 (51%) | 9 (20%) | 5 (11%) | 33 | 10 (30%) | 16 (49%) | 6 (18%) | 1 (3%) |
| Cough | 113 | 45 (40%) | 46 (41%) | 21 (19%) | 1 (1%) | 63 | 21 (33%) | 29 (46%) | 12 (19%) | 1 (2%) | 50 | 24 (48%) | 17 (34%) | 9 (18%) | 0 (0%) |
| Chest pain | 74 | 27 (37%) | 30 (41%) | 13 (20%) | 4 (5%) | 42 | 12 (29%) | 20 (48%) | 8 (19%) | 2 (5%) | 32 | 15 (47%) | 10 (31%) | 5 (16%) | 2 (6%) |
| Skin rash | 98 | 29 (30%) | 44 (45%) | 19 (19%) | 6 (6%) | 57 | 16 (28%) | 28 (49%) | 9 (16%) | 4 (7%) | 41 | 13 (32%) | 16 (39%) | 10 (24%) | 2 (5%) |
| Phlegm | 90 | 35 (39%) | 36 (40%) | 18 (20%) | 1 (1%) | 54 | 21 (39%) | 23 (43%) | 9 (17%) | 1 (2%) | 36 | 14 (39%) | 13 (36%) | 9 (25%) | 0 (0%) |
| Vision problems | 115 | 52 (45%) | 50 (44%) | 10 (9%) | 3 (3%) | 67 | 28 (42%) | 32 (48%) | 6 (9%) | 1 (2%) | 48 | 24 (50%) | 18 (38%) | 4 (8%) | 2 (4%) |
| Anosmia | 86 | 30 (35%) | 33 (38%) | 18 (21%) | 5 (6%) | 56 | 18 (32%) | 23 (41%) | 11 (20%) | 4 (7%) | 30 | 12 (40%) | 10 (33%) | 7 (23%) | 1 (3%) |
| Joint pain | 135 | 35 (26%) | 45 (40%) | 39 (29%) | 7 (5%) | 73 | 20 (27%) | 30 (41%) | 20 (27%) | 3 (4%) | 62 | 15 (24%) | 24 (39%) | 19 (31%) | 4 (7%) |
| Hoarseness | 92 | 36 (39%) | 42 (46%) | 12 (13%) | 2 (2%) | 42 | 15 (36%) | 22 (52%) | 5 (12%) | 0 (0%) | 50 | 21 (42%) | 20 (40%) | 7 (14%) | 2 (4%) |
| Ageusia | 81 | 30 (37%) | 34 (42%) | 15 (19%) | 2 (3%) | 52 | 14 (27%) | 24 (46%) | 13 (25%) | 1 (2%) | 29 | 16 (55%) | 10 (35%) | 2 (7%) | 1 (3%) |
| Stool problems | 63 | 18 (29%) | 19 (30%) | 19 (30%) | 7 (11%) | 33 | 9 (27%) | 10 (30%) | 11 (33%) | 3 (9%) | 30 | 9 (30%) | 9 (30%) | 8 (27%) | 4 (13%) |
| Hearing problems | 82 | 25 (31%) | 44 (54%) | 12 (15%) | 1 (1%) | 54 | 13 (24%) | 32 (59%) | 8 (15%) | 1 (2%) | 28 | 12 (43%) | 12 (43%) | 4 (14%) | 0 (0%) |
| Anxiety/ nightmares | 49 | 14 (29%) | 17 (35%) | 14 (29%) | 4 (8%) | 20 | 6 (30%) | 7 (35%) | 4 (20%) | 3 (15%) | 29 | 8 (28%) | 10 (35%) | 10 (35%) | 1 (3%) |
| Claudication | 35 | 11 (31%) | 18 (51%) | 5 (14%) | 1 (3%) | 15 | 3 (20%) | 9 (60%) | 2 (13%) | 1 (7%) | 20 | 8 (40%) | 9 (45%) | 3 (15%) | 0 (0%) |
| Miction problems | 50 | 10 (20%) | 23 (46%) | 15 (30%) | 2 (4%) | 25 | 5 (20%) | 10 (40%) | 9 (36%) | 1 (4%) | 25 | 5 (20%) | 13 (52%) | 6 (24%) | 1 (4%) |

Data are presented as n (%). At the 2-year study visit, patients with symptoms were asked to indicate the severity of each symptom. The severity of the symptom dyspnea is not included as it was assessed with the Modified Medical Research Council Dyspnea Scale. *ICU* Intensive Care Unit

**Table S3A.** Categorical outcomes on the mMRC dyspnea scale, IFIS, and recovery status questionnaires in patients with COVID-19 up to 2 years after hospital discharge.

|  | **Total** | | | | **Non-ICU** | | | | **ICU** | | | |
| --- | --- | --- | --- | --- | --- | --- | --- | --- | --- | --- | --- | --- |
|  | **3**  **months** | **6**  **months** | **1**  **year** | **2**  **years** | **3**  **months** | **6**  **months** | **1**  **year** | **2**  **years** | **3**  **months** | **6**  **months** | **1**  **year** | **2**  **years** |
| **mMRC dyspnea scale, *n*** | *433* | *484* | *473* | *466* | *270* | *289* | *285* | *278* | *163* | *195* | *188* | *188* |
| No dyspnea | 79 (18%) | 103 (21%) | 104 (22%) | 96 (21%) | 45 (17%) | 63 (22%) | 64 (22%) | 64 (23%) | 34 (21%) | 40 (21%) | 40 (21%) | 32 (17%) |
| Grade 0 | 179 (41%) | 207 (43%) | 206 (44%) | 183 (39%) | 108 (40%) | 116 (40%) | 114 (40%) | 101 (36%) | 71 (44%) | 91 (47%) | 92 (49%) | 82 (44%) |
| Grade 1 | 86 (20%) | 90 (19%) | 88 (19%) | 96 (21%) | 60 (22%) | 58 (20%) | 64 (23%) | 55 (20%) | 26 (16%) | 32 (16%) | 24 (13%) | 41 (22%) |
| Grade 2 | 62 (14%) | 65 (13%) | 60 (13%) | 62 (13%) | 38 (14%) | 42 (15%) | 38 (13%) | 42 (15%) | 24 (15%) | 23 (12%) | 22 (12%) | 20 (11%) |
| Grade 3 | 18 (4%) | 14 (3%) | 8 (2%) | 23 (5%) | 13 (5%) | 9 (3%) | 3 (1%) | 13 (5%) | 5 (3%) | 5 (3%) | 5 (3%) | 10 (5%) |
| Grade 4 | 9 (2%) | 5 (1%) | 7 (1%) | 6 (1%) | 6 (2%) | 1 (0.3%) | 2 (1%) | 3 (1%) | 3 (2%) | 4 (2%) | 5 (3%) | 3 (2%) |
| **IFIS, *n*** | *451* | *491* | *484* | *447* | *275* | *297* | *291* | *264* | *176* | *194* | *193* | *183* |
| Very poor | 22 (5%) | 7 (1%) | 13 (3%) | 15 (3%) | 12 (4%) | 5 (2%) | 8 (3%) | 11 (4%) | 10 (6%) | 2 (1%) | 5 (3%) | 4 (2%) |
| Poor | 106 (24%) | 78 (16%) | 74 (15%) | 66 (15%) | 58 (21%) | 47 (16%) | 39 (13%) | 39 (15%) | 48 (27%) | 31 (16%) | 34 (18%) | 27 (15%) |
| Average | 193 (43%) | 226 (46%) | 217 (45%) | 191 (43%) | 124 (45%) | 136 (46%) | 134 (46%) | 105 (40%) | 69 (39%) | 90 (46%) | 83 (43%) | 86 (47%) |
| Good | 114 (25%) | 151 (31%) | 152 (31%) | 150 (34%) | 69 (25%) | 89 (30%) | 96 (33%) | 93 (35%) | 45 (26%) | 62 (32%) | 56 (29%) | 57 (31%) |
| Very good | 17 (4%) | 30 (6%) | 32 (6%) | 25 (6%) | 13 (5%) | 20 (7%) | 17 (6%) | 16 (6%) | 4 (2%) | 10 (4%) | 15 (8%) | 9 (5%) |
| **Recovery status, *n*** | *159* | *300* | *418* | *443* | *90* | *184* | *225* | *260* | *69* | *116* | *193* | *183* |
| Not recovered at all | 1 (1%) | 2 (1%) | 3 (2%) | 8 (2%) | 1 (1%) | 2 (1%) | 2 (1%) | 7 (3%) | 0 (0%) | 0 (0%) | 1 (1%) | 1 (1%) |
| Somewhat recovered | 24 (15%) | 18 (6%) | 21 (5%) | 22 (5%) | 14 (15%) | 13 (7%) | 11 (4%) | 13 (5%) | 10 (15%) | 5 (4%) | 10 (5%) | 9 (5%) |
| Half recovered | 34 (21%) | 59 (20%) | 74 (17%) | 73 (17%) | 21 (23%) | 27 (15%) | 40 (16%) | 37 (14%) | 13 (19%) | 32 (28%) | 34 (18%) | 36 (20%) |
| Mostly recovered | 83 (52%) | 169 (56%) | 248 (55%) | 219 (49%) | 42 (47%) | 100 (54%) | 136 (53%) | 123 (47%) | 41 (59%) | 69 (60%) | 112 (58%) | 96 (53%) |
| Completely recovered | 17 (11%) | 52 (17%) | 102 (23%) | 121 (27%) | 12 (13%) | 42 (23%) | 66 (26%) | 80 (31%) | 5 (7%) | 10 (9%) | 36 (19%) | 41 (22%) |

Data are presented as n (%). The Modified Medical Research Council Dyspnea contains the following grades: grade 0, dyspnea only with strenuous exercise; grade 1, dyspnea when hurrying or walking up a slight hill; grade 2, walks slower than people of the same age because of dyspnea or has to stop for breath when walking at own pace; grade 3, stops for breath after walking 100 meters or after a few minutes; and grade 4, too dyspneic to leave the house or breathless when dressing. *IFIS* International Fitness Scale, *ICU* Intensive Care Unit

**Table S3B.** Domain scores of the EQ-5D-5L and SF-36 questionnaires in patients with COVID-19 up to 2 years after hospital discharge.

|  | **Total** | | | | | **Non-ICU** | | | | **ICU** | | | **Overall comparison,**  **ICU vs non-ICU** | | | **Interaction ICU * Time** |
| --- | --- | --- | --- | --- | --- | --- | --- | --- | --- | --- | --- | --- | --- | --- | --- | --- |
|  | **3**  **months** | **6**  **months** | **1**  **year** | **2**  **years** | **P value** | **3**  **months** | **6**  **months** | **1**  **year** | **2**  **years** | **3**  **months** | **6**  **months** | **1**  **year** | | **2**  **years** | **P value** | **P value** |
| **EQ-5D-5L, n** | *442* | *482* | *479* | *437* |  | *274* | *288* | *289* | *260* | *168* | *194* | *190* | | *177* |  |  |
| Pain and discomfort | 293 (66%) | 303 (63%) | 284 (59%)^#^ | 275 (63%) | 0.008 | 175 (64%) | 175 (61%) | 163 (57%) | 159 (61%) | 118 (70%) | 128 (66%) | 120 (63%) | | 116 (66%) | 0.15 | 0.85 |
| Anxiety or depression | 157 (36%) | 157 (33%) | 142 (30%)^#^ | 123 (28%) | 0.006 | 101 (37%) | 92 (32%) | 86 (30%) | 76 (29%) | 56 (33%) | 65 (34%) | 56 (30%) | | 47 (27%) | 0.75 | 0.23 |
| Mobility problem | 217 (49%) | 202 (42%)* | 181 (38%)*^#^ | 187 (43%) | **<0.001** | 120 (44%) | 112 (39%) | 105 (36%) | 107 (41%) | 97 (58%) | 90 (46%) | 76 (40%) | | 80 (45%) | 0.05 | 0.12 |
| Personal care problem | 69 (16%) | 38 (8%)* | 41 (9%)^#^ | 39 (9%) | **<0.001** | 33 (12%) | 19 (7%) | 17 (6%) | 20 (8%) | 36 (21%) | 19 (10%) | 23 (12%) | | 19 (11%) | 0.02 | 0.33 |
| Usual activity problem | 257 (58%) | 236 (49%)* | 209 (44%)*^#^ | 180 (41%)^ꬹ^ | **<0.001** | 151 (55%) | 138 (48%) | 119 (42%) | 107 (41%) | 106 (63%) | 98 (51%) | 89 (47%) | | 73 (41%) | 0.25 | 0.16 |
| **SF-36, n** | *434* | *481* | *476* | *441* |  | *274* | *292* | *286* | *262* | *161* | *190* | *191* | | *179* |  |  |
| Physical functioning | 64.1 (25.9) | 70.0 (23.9)* | 71.8 (24.6)*^#^ | 71.9 (24.6)^ꬹ^ | **<0.001** | 65.6 (26.5) | 71.1 (24.2) | 72.1 (24.2) | 73.2 (24.9) | 61.6 (24.9) | 68.3 (23.4) | 71.4 (25.2) | | 70.2 (24.1) | 0.32 | 0.03 |
| Physical role impairment | 38.6 (43.0) | 47.3 (43.8)* | 57.6 (42.5)*^#^ | 62.6 (41.8)^ꬹ^ | **<0.001** | 41.3 (43.6) | 51.5 (43.3) | 61.3 (41.9) | 65.8 (41.1) | 34.0 (41.6) | 40.9 (43.9) | 52.1 (43.0) | | 57.9 (42.5) | 0.007 | 0.77 |
| Emotional role impairment | 62.3 (43.8) | 69.2 (40.9)* | 7`2.8 (38.3)^#^ | 77.1 (36.4)^ꬹ^ | **<0.001** | 62.8 (43.5) | 68.2 (41.8) | 74.6 (37.6) | 76.4 (36.7) | 61.5 (44.3) | 70.7 (39.7) | 70.2 (39.1) | | 78.2 (36.1) | 0.83 | 0.27 |
| Vitality | 55.1 (22.2) | 57.9 (22.0)* | 69.5 (21.8)^#^ | 59.4 (22.0) | **<0.001** | 53.8 (22.3) | 55.9 (22.2) | 58.8 (21.5) | 58.9 (22.0) | 57.3 (21.8) | 61.0 (21.5) | 60.6 (22.4) | | 60.2 (21.9) | 0.17 | 0.11 |
| Mental health | 74.5 (19.3) | 76.0 (18.2) | 76.5 (18.7)^#^ | 76.7 (17.3) | 0.02 | 73.3 (19.3) | 74.3 (18.2) | 75.9 (17.9) | 77.0 (16.5) | 76.6 (19.1) | 78.5 (17.9) | 77.5 (19.9) | | 76.3 (18.4) | 0.26 | 0.01 |
| Social functioning | 68.7 (28.4) | 74.8 (25.2)* | 77.0 (24.9)*^#^ | 77.9 (23.7) | **<0.001** | 68.4 (28.0) | 74.1 (25.7) | 78.0 (23.5) | 78.0 (23.7) | 69.1 (29.2) | 75.9 (24.5) | 75.5 (26.7) | | 77.8 (23.8) | 0.87 | 0.49 |
| Bodily pain | 69.2 (25.8) | 72.2 (25.9) | 74.3 (25.0)*^#^ | 74.2 (25.1) | **<0.001** | 70.1 (25.5) | 73.4 (25.0) | 75.8 (24.7) | 74.7 (25.6) | 67.7 (26.4) | 70.4 (27.1) | 72.1 (25.2) | | 73.5 (24.5) | 0.33 | 0.42 |
| General health | 53.6 (22.2) | 56.6 (22.2)* | 55.8 (22.3) | 53.5 (23.0)* | **<0.001** | 53.0 (22.5) | 55.7 (22.7) | 55.2 (22.3) | 53.6 (24.1) | 54.5 (21.8) | 57.9 (21.4) | 56.8 (22.3) | | 53.3 (21.4) | 0.35 | 0.38 |
| The data comprise raw test outcomes and are presented as n (%) for EQ-5D-5L and mean (standard deviation) for SF-36. Domains of EQ-5D-5L were dichotomized into experiencing problems (slight, moderate, severe, or unable/extreme) vs not experiencing problems. P values are obtained from Generalized Estimating Equations analysis, a P value less than 0.004 was considered statistically significant and is indicated in bold. In the total cohort, ^*^ indicates a significant difference as compared to the previous study visit, ^#^ a significant difference between the 3-month and 1-year study visits, and ^ꬹ^ between the 6-month and 2-year study visits. *EQ-5D-5L* 5-level EuroQoL-5D questionnaire, *SF-36* 36-item Short Form Health Survey, *ICU* Intensive Care Unit | | | | | | | | | | | | | | | | |

**Table S4.** Trajectories of mental health and physical function up to 2 years after hospitalization within ICU- and non-ICU-treated COVID-19 patients.

|  | **3**  **months** | **6**  **months** | **1**  **year** | **2**  **years** | **Mean difference 3-6 months**  **(95% CI), P value** | **Mean difference 6 months-1 year**  **(95% CI), P value** | **Mean difference 1-2 year**  **(95% CI), P value** |
| --- | --- | --- | --- | --- | --- | --- | --- |
| **Mental health** |  |  |  |  |  |  |  |
| **Cognitive failures,**  **total CFQ score** |  |  |  |  |  |  |  |
| ICU | 28.6 (1.3) | 30.1 (1.2) | 31.0 (1.3) | 32.4 (1.4) | 1.5 (-0.24 to 3.3), 0.09 | 0.9 (-0.6 to 2.4), 0.23 | 1.4 (-0.06 to 2.9), 0.06 |
| Non-ICU | 32.9 (1.1) | 32.5 (1.1) | 33.3 (1.1) | 32.2 (1.1) | -0.4 (-1.7 to 0.9), 0.56 | 0.8 (-0.3 to 2.0), 0.16 | -1.2 (-2.4 to 0.09), 0.07 |
| **PTSD, total IES-R score** |  |  |  |  |  |  |  |
| ICU | 17.2 (1.1) | 14.6 (0.9) | 15.7 (1.0) | 15.1 (1.0) | -2.6 (-4.0 to -1.1), <0.001 | 1.2 (0.01 to 2.3), 0.05 | -0.6 (-1.8 to 0.5), 0.29 |
| Non-ICU | 14.7 (0.8) | 12.9 (0.7) | 11.7 (0.7) | 10.5 (0.7) | -1.8 (-2.7 to -0.9), <0.001 | -1.2 (-2.1 to -0.4), 0.003 | -1.2 (-2.0 to -0.5), 0.002 |
| **Anxiety, HADS-A ≥11** |  |  |  |  |  |  |  |
| ICU | 0.1 (0.02) | 0.1 (0.02) | 0.1 (0.03) | 0.2 (0.03) | 0.003 (-0.05 to 0.05), 0.92 | 0.03 (-0.01 to 0.07), 0.16 | 0.004 (-0.04 to 0.04), 0.85 |
| Non-ICU | 0.1 (0.02) | 0.1 (0.02) | 0.1 (0.02) | 0.1 (0.02) | -0.04 (-0.10 to 0.02), 0.23 | -0.01 (-0.05 to 0.02), 0.48 | -0.03 (-0.09 to 0.02), 0.27 |
| **Depression, HADS-D ≥11** |  |  |  |  |  |  |  |
| ICU | 0.1 (0.02) | 0.1 (0.02) | 0.1 (0.02) | 0.1 (0.03) | -0.03 (-0.07 to 0.02), 0.22 | 0.05 (0.01 to 0.1), 0.015 | 0.01 (-0.04 to 0.05), 0.78 |
| Non-ICU | 0.1 (0.02) | 0.1 (0.02) | 0.1 (0.02) | 0.1 (0.02) | 0.004 (-0.05 to 0.04), 0.85 | -0.02 (-0.06 to 0.01), 0.21 | -0.01 (-0.04 to 0.03), 0.73 |
| **Physical function** |  |  |  |  |  |  |  |
| **Aerobic capacity** |  |  |  |  |  |  |  |
| **6MWT, 6MWD %pred** |  |  |  |  |  |  |  |
| ICU | 85.2 (1.5) | 90.4 (1.5) | 92.9 (1.3) | 96.7 (1.3) | 5.1 (2.9 to 7.4), <0.001 | 2.5 (0.7 to 4.3), 0.008 | 3.8 (2.4 to 5.3),<0.001 |
| Non-ICU | 87.7 (1.2) | 89.4 (1.2) | 91.0 (1.2) | 91.4 (1.3) | 1.7 (0.02 to 3.4), 0.05 | 1.6 (0.2 to 3.1), 0.03 | 0.4 (-1.0 to 1.8), 0.57 |
| **1MSTST, number of STS repetitions %pred** |  |  |  |  |  |  |  |
| ICU | 63.8 (1.7) | 73.9 (1.8) | 78.8 (2.0) | 83.8 (2.0) | 10.1 (7.3 to 12.9), <0.001 | 4.9 (2.2 to 7.7), <0.001 | 5.0 (2.4 to 7.6), <0.001 |
| Non-ICU | 67.8 (1.5) | 71.4 (1.5) | 74.2 (1.6) | 79.8 (1.7) | 3.5 (1.5 to 5.6), 0.001 | 2.9 (0.9 to 4.9), 0.005 | 5.6 (3.4 to 7.9), <0.001 |
| **Muscle strength** |  |  |  |  |  |  |  |
| **Maximum HGS %pred** |  |  |  |  |  |  |  |
| ICU | 84.4 (1.5) | 94.8 (1.5) | 105.4 (1.4) | 106.4 (1.5) | 10.3 (8.3 to 12.4), <0.001 | 10.7 (9.0 to 12.4), <0.001 | 0.9 (-1.1 to 3.0), 0.37 |
| Non-ICU | 95.3 (1.3) | 103.7 (1.4) | 106.5 (1.4) | 107.3 (1.4) | 8.3 (6.5 to 10.2), <0.001 | 2.8 (1.2 to 4.5), 0.001 | 0.8 (-1.0 to 2.5), 0.39 |

The data are presented as estimated means (standard errors) and only for Anxiety and Depression as estimated proportions (standard errors), obtained from Generalized Estimating Equations analysis. For mental health, estimated means and estimated proportions are corrected for age and sex. For physical outcomes, data are presented as percentage of normative values reached in 6MWT, 1MSTST, and HGS. We used normative values in 6MWT that are calculated using sex-, age-, height-, and weight-stratified equations described by Enright and Sherill,^44^ in 1MSTST using sex- and age-stratified reference values described by Strassman and colleagues,^45^ and in HGS using sex- and age-stratified reference values described by Dodds and colleagues.^46^ *CFQ* Cognitive Failures Questionnaire, *IES-R* Impact of Event Scale-Revised, *6MWT* 6 Min Walk Test, *1MSTST* 1 Min Sit-To-Stand Test, *HGS* Handgrip Strength, *%pred* percentage of normative values, *ICU* Intensive Care Unit

**Table S5.** Outcomes of objectively assessed cognitive and physical function in COVID-19 patients up to 2 years after hospital discharge.

|  | **Total** | | | | | **Non-ICU** | | | | **ICU** | | | **Overall comparison,**  **ICU vs non-ICU** | | | | **Interaction ICU * Time** | | |
| --- | --- | --- | --- | --- | --- | --- | --- | --- | --- | --- | --- | --- | --- | --- | --- | --- | --- | --- | --- |
|  | **3**  **months** | **6**  **months** | **1**  **year** | **2**  **year** | **P value** | **3**  **months** | **6**  **months** | **1**  **year** | **2**  **year** | **3**  **months** | **6**  **months** | **1**  **year** | | **2**  **year** | **P value** | **P value** | | |  |
| **Cognitive function** |  |  |  |  |  |  |  |  |  |  |  |  | |  |  |  | | |  |
| MoCA, *n* | *414* | *484* | *485* | *464* |  | *262* | *288* | *282* | *269* | *152* | *196* | *203* | | *195* |  |  | | |  |
| MoCA, total score LOCF | 25.7 (2.8) | 26.2 (2.6)* | 26.4 (2.4)*^#^ | 26.8 (2.1)*^ꬹ^ | **<0.001** | 25.5 (2.9) | 26.2 (2.6) | 26.4 (2.4) | 26.7 (2.1) | 26.1 (2.6) | 26.2 (2.7) | 26.4 (2.5) | | 26.8 (2.2) | 0.23 | 0.89 | | |  |
| MoCA, <26 LOCF | 165 (40%) | 135 (28%)* | 101 (21%)*^#^ | 57 (12%)*^ꬹ^ | **<0.001** | 112 (43%) | 79 (27%) | 58 (21%) | 33 (12%) | 53 (35%) | 56 (29%) | 43 (21%) | | 24 (12%) | 0.68 | 0.70 | | |  |
| **Physical function** |  |  |  |  |  |  |  |  |  |  |  |  | |  |  |  | | |  |
| **Aerobic capacity** |  |  |  |  |  |  |  |  |  |  |  |  | |  |  |  | | |  |
| *6MWT, n* | *399* | *454* | *424* | *373* |  | *247* | *263* | *245* | *218* | *152* | *191* | *179* | | *155* |  |  | | |  |
| 6MWD, m | 482.0 (114.4) | 495.8 (115.6)* | 506.3 (107.6)*^#^ | 511.5 (109.4)^ꬹ^ | **<0.001** | 488.0 (115.3) | 496.9 (113.6) | 505.0 (111.5) | 501.1 (113.1) | 472.2 (112.7) | 494.4 (118.6) | 508.0 (102.2) | | 526.1 (102.6) | 0.53 | **<0.001** | | |  |
| 6MWD, %pred | 88.0 (19.6) | 90.5 (20.1)* | 93.0 (18.7)*^#^ | 94.6 (19.1)*^ꬹ^ | **<0.001** | 88.5 (19.4) | 90.5 (19.6) | 92.0 (19.4) | 92.6 (19.9) | 87.3 (19.9) | 90.7 (20.9) | 94.3 (17.7) | | 97.3 (17.7) | 0.42 | **<0.001** | | |  |
| 6MWD, <LLN | 81 (21%) | 73 (16%)* | 61 (15%)^#^ | 38 (10%)*^ꬹ^ | **<0.001** | 49 (21%) | 41 (16%) | 42 (17%) | 29 (13%) | 32 (23%) | 32 (17%) | 19 (11%) | | 9 (6%) | 0.26 | **0.005** | | |  |
| Desaturation ≥ 4% | 99 (25%) | 97 (22%) | 86 (21%) | 69 (19%) | 0.09 | 52 (21%) | 40 (16%) | 41 (17%) | 23 (11%) | 47 (32%) | 57 (32%) | 45 (26%) | | 46 (30%) | **<0.001** | 0.07 | | |  |
| *1MSTST, n* | *418* | *482* | *495* | *414* |  | *264* | *290* | *276* | *245* | *150* | *205* | *206* | | *173* |  |  | | |  |
| Number of STS repetitions | 252 (10.0) | 27.1 (10.3)* | 28.2 (10.9)*^#^ | 30.0 (11.0)*^ꬹ^ | **<0.001** | 25.5 (10.0) | 26.6 (9.9) | 27.3 (10.3) | 29.3 (11.1) | 24.6 (9.8) | 27.8 (10.9) | 29.4 (11.6) | | 31.1 (10.9) | 0.15 | **<0.001** | | |  |
| STS repetitions, %pred | 67.5 (24.8) | 73.3 (26.5)* | 77.0 (28.8)*^#^ | 82.7 (28.8)*^ꬹ^ | **<0.001** | 68.8 (25.4) | 72.4 (25.5) | 75.0 (27.4) | 81.4 (29.2) | 65.4 (23.7) | 74.4 (27.8) | 79.7 (30.4) | | 84.6 (28.2) | 0.41 | **<0.001** | | |  |
| **Muscle strength** |  |  |  |  |  |  |  |  |  |  |  |  | |  |  |  | | |  |
| *HGS, n* | *438* | *496* | *514* | *446* |  | *279* | *302* | *285* | *259* | *167* | *212* | *211* | | *179* |  |  | | |  |
| Maximum HGS, kg | 36.1 (12.9) | 39.0 (13.6)* | 41.1 (13.9)*^#^ | 41.4 (14.1)^ꬹ^ | **<0.001** | 37.1 (13.5) | 39.4 (14.4) | 40.6 (14.6) | 40.6 (14.5) | 34.3 (11.6) | 38.3 (12.3) | 41.7 (12.9) | | 42.5 (13.4) | 0.55 | **<0.001** | | |  |
| Maximum HGS, %pred | 92.3 (23.5) | 100.6 (24.4)* | 106.8 (23.9)*^#^ | 108.0 (24.0)^ꬹ^ | **<0.001** | 96.7 (23.8) | 104.2 (24.9) | 108.3 (25.1) | 109.2 (25.5) | 84.8 (21.0) | 95.4 (22.8) | 104.9 (22.0) | | 106.3 (21.7) | **0.002** | **<0.001** | | |  |
| **Mobility** |  |  |  |  |  |  |  |  |  |  |  |  | |  |  |  | | |  |
| *DEMMI, n* | *432* | *487* | *500* | *424* |  | *265* | *292* | *279* | *256* | *159* | *208* | *208* | | *176* |  |  | | |  |
| DEMMI, total score | 87.8 (13.3) | 88.7 (13.0) | 89.5 (12.2)^#^ | 89.2 (12.6) | 0.03 | 87.6 (14.2) | 88.0 (13.5) | 89.4 (12.8) | 89.0 (13.8) | 88.0 (11.8) | 89.8 (12.1) | 89.8 (11.3) | | 89.5 (10.6) | 0.21 | 0.20 | | |  |
| The data comprise raw test outcomes and are presented as mean (standard deviation) or n (%). P values are obtained from Generalized Estimating Equations analysis, a P value less than 0.01 was considered statistically significant and is indicated in bold. In the total cohort, ^*^ indicates a significant difference as compared to the previous study visit, ^#^ a significant difference between the 3-month and 1-year study visits, and ^ꬹ^ between the 6-month and 2-year study visits. Normative values in 6MWT are calculated using sex-, age-, height-, and weight-stratified equations described by Enright and Sherill,^44^ in 1MSTST using sex- and age-stratified reference values described by Strassman and colleagues,^45^ and in HGS using sex- and age-stratified reference values described by Dodds and colleagues.^46^ *ICU* Intensive Care Unit, *MoCA* Montreal Cognitive Assessment, *LOCF* Last Observation Carried Forward, *6MWT* 6 Min Walk Test, *6MWD* 6 Min Walk Distance, *LLN* Lower Limit of Normal, *1MSTST* 1 Min Sit-To-Stand Test, *HGS* Handgrip Strength, *DEMMI* de Morton Mobility Index | | | | | | | | | | | | | | | | | |  |  |

**Table S6.** Pulmonary function testing and radiologic outcomes in the total cohort up to 2 years after hospitalization for COVID-19.

|  | **6 weeks** | | **3 months** | | **6 months** | | **1 year** | | **2 years** | |
| --- | --- | --- | --- | --- | --- | --- | --- | --- | --- | --- |
| **Spirometry** | ***n*** |  | ***n*** |  | ***n*** |  | ***n*** |  | ***n*** |  |
| FVC, L | *243* | 3.5 (1.0) | *349* | 3.6 (1.0) | *144* | 3.6 (1.1) | *106* | 3.4 (1.0) | *53* | 3.5 (0.9) |
| FVC% predicted | *243* | 86.5 (16.0) | *351* | 89.6 (17.1) | *149* | 88.9 (18.4) | *106* | 86.7 (17.6) | *53* | 88.2 (16.8) |
| FVC% < LLN, n | *242* | 60 (25%) | *333* | 71 (21%) | *131* | 30 (23%) | *101* | 25 (25%) | *51* | 12 (24%) |
| FEV_1_, L | *247* | 2.8 (0.8) | *389* | 2.9 (0.8) | *154* | 2.8 (0.8) | *108* | 2.6 (0.8) | *55* | 2.7 (0.8) |
| FEV_1_% predicted | *247* | 88.8 (15.6) | *391* | 91.0 (16.7) | *158* | 88.3 (18.5) | *107* | 86.0 (19.5) | *55* | 87.3 (18.6) |
| FEV_1_% < LLN, n | *245* | 46 (19%) | *372* | 65 (18%) | *140* | 30 (21%) | *103* | 23 (22%) | *52* | 10 (19%) |
| **Gas exchange** |  |  |  |  |  |  |  |  |  |  |
| DlCO_c_, mmol/(min*kPa) | *230* | 6.2 (1.9) | *352* | 6.6 (2.0) | *122* | 6.2 (1.7) | *86* | 6.2 (1.8) | *30* | 5.8 (14.9) |
| DlCO_c_% predicted | *229* | 72.9 (16.3) | *357* | 75.5 (16.9) | *129* | 73.0 (14.3) | *86* | 73.8 (16.1) | *30* | 68.8 (14.9) |
| DlCO_c_% < LLN, n | *225* | 131 (58%) | *340* | 166 (49%) | *111* | 72 (65%) | *79* | 42 (53%) | *27* | 18 (67%) |
| **Chest X-ray abnormalities, n** |  | *419* |  | *127* |  | *64* |  | *48* |  | *40* |
| Normal |  | 156 (37%) |  | 68 (54%) |  | 39 (61%) |  | 29 (60%) |  | 22 (55%) |
| Moderate |  | 162 (39%) |  | 42 (33%) |  | 18 (28%) |  | 12 (25%) |  | 15 (38%) |
| Severe |  | 101 (24%) |  | 17 (13%) |  | 7 (11%) |  | 7 (15%) |  | 3 (8%) |
| **Chest CT scan abnormalities, n** |  | *26* |  | *250* |  | *121* |  | *72* |  | *37* |
| Normal |  | 4 (15%) |  | 28 (11%) |  | 7 (6%) |  | 3 (4%) |  | 2 (5%) |
| Ground-glass opacities |  | 16 (62%) |  | 153 (61%) |  | 67 (55%) |  | 32 (44%) |  | 23 (62%) |
| Moderate |  | 5 (19%) |  | 102 (41%) |  | 51 (42%) |  | 25 (35%) |  | 15 (41%) |
| Severe |  | 11 (42%) |  | 51 (20%) |  | 16 (13%) |  | 7 (10%) |  | 8 (22%) |
| Bronchiectasis or bronchiolectasis |  | 6 (23%) |  | 73 (29%) |  | 44 (28%) |  | 23 (37%) |  | 9 (24%) |
| Moderate |  | 2 (8%) |  | 46 (18%) |  | 23 (19%) |  | 14 (19%) |  | 6 (16%) |
| Severe |  | 4 (15%) |  | 27 (11%) |  | 11 (9%) |  | 9 (13%) |  | 3 (8%) |
| Consolidation |  | 6 (23%) |  | 31 (12%) |  | 16 (13%) |  | 6 (8%) |  | 5 (14%) |
| Reticulation/fibrosis |  | 6 (23%) |  | 80 (32%) |  | 44 (36%) |  | 30 (42%) |  | 11 (30%) |
| Subpleural lines and bands |  | 5 (19%) |  | 57 (23%) |  | 38 (31%) |  | 16 (22%) |  | 11 (30%) |

Data are presented as the mean (standard deviation) or n (%). *FVC* Forced Vital Capacity, *LLN* Lower Limit of Normal, *FEV_1_* Forced Expiratory Volume in 1s, *DLCOc* Diffusing Capacity of the Lung for Carbon monoxide adjusted for hemoglobin

**Table S7. Pulmonary function testing and radiological outcomes in 55 patients with initial poor pulmonary recovery who continued follow-up up to 2 years after hospitalization for COVID-19.**

|  | **6 weeks** | | **3 months** | **6 months** | **1 year** | **2 years** | **P value** |
| --- | --- | --- | --- | --- | --- | --- | --- |
| **Spirometry** |  | |  |  |  |  |  |
| FVC, L | 2.7 (1.0) | | 3.1 (0.9) | 3.3 (0.9) | 3.3 (0.9) | 3.5 (0.9) | **<0.001** |
| FVC% predicted | 75.6 (19.0) | | 79.1 (16.9) | 82.3 (17.6) | 86.2 (17.8) | 88.2 (16.8) | **<0.001** |
| FVC *z*-score | -1.7 (1.3) | | -1.5 (1.1) | -1.2 (1.2) | -1.0 (1.2) | -0.81 (1.1) | **<0.001** |
| FVC% < LLN, *n* | 14/24 (58%) | | 21/40 (53%) | 12/31 (39%) | 11/35 (31%) | 12/51 (24%) | **0.004** |
| FEV_1_, L | 2.2 (0.6) | | 2.5 (0.7) | 2.5 (0.7) | 2.6 (0.8) | 2.7 (0.8) | **<0.001** |
| FEV_1_% predicted | 75.0 (16.5) | | 78.8 (15.1) | 81.6 (15.6) | 86.2 (16.7) | 87.3 (18.6) | **<0.001** |
| FEV_1_ *z*-score | -1.6 (1.0) | | -1.4 (0.9) | -1.2 (0.9) | -0.89 (1.0) | -0.82 (1.1) | **<0.001** |
| FEV_1_% < LLN, *n* | 12/24 (50%) | | 17/43 (40%) | 9/32 (28%) | 9/37 (24%) | 10/52 (19%) | 0.007 |
| **Gas exchange** |  | |  |  |  |  |  |
| DlCO_c_, mmol/(min*kPa) | 4.8 (1.5) | | 5.3 (1.6) | 5.4 (1.1) | 5.8 (1.9) | 5.8 (1.6) | **<0.001** |
| DlCO_c_% predicted | 59.9 (14.6) | | 65.0 (15.0) | 65.1 (10.4) | 68.4 (15.3) | 68.8 (14.9) | **<0.001** |
| DlCO_c_ *z*-score | -3.1 (1.4) | | -2.6 (1.4) | -2.5 (0.9) | -2.3 (1.2) | -2.3 (1.4) | **<0.001** |
| DlCO_c_% < LLN, *n* | 17/21 (81%) | | 32/40 (80%) | 24/26 (92%) | 23/32 (72%) | 18/27 (67%) | 0.02 |
| **Chest X-ray abnormalities** | 38 | | 12 | 6 | 10 | 21 | 0.02 |
| Normal | 11 (29%) | | 7 (58%) | 4 (67%) | 5 (50%) | 10 (48%) |  |
| Moderate | 9 (24%) | | 4 (33%) | 2 (33%) | 5 (50%) | 10 (48%) |  |
| Severe | 18 (47%) | | 1 (8%) | 0 (0%) | 0 (0%) | 1 (5%) |  |
| **Chest CT scan abnormalities** | | 6 | 34 | 26 | 19 | 19 |  |
| Normal | 0 (0%) | | 0 (0%) | 0 (0%) | 0 (0%) | 0 (0%) | NA |
| GGO |  | |  |  |  |  |  |
| Moderate | 0 (0%) | | 12 (22%) | 7 (13%) | 7 (13%) | 9 (16%) | 0.4 |
| Severe | 3 (6%) | | 10 (18%) | 4 (7%) | 2 (4%) | 4 (7%) | 0.008 |
| Bronchiectasis or  bronchiolectasis |  | |  |  |  |  |  |
| Moderate | 0 (0%) | | 5 (9%) | 3 (6%) | 3 (6%) | 4 (7%) | 0.7 |
| Severe | 1 (2%) | | 5 (9%) | 3 (6%) | 5 (9%) | 2 (4%) | 0.3 |
| Consolidation | 3 (6%) | | 6 (11%) | 4 (7%) | 2 (4%) | 2 (4%) | 0.3 |
| Reticulation/fibrosis | 3 (6%) | | 19 (35%) | 15 (27%) | 12 (22%) | 8 (15%) | 0.01 |
| Subpleural lines and  bands | 0 (0%) | | 13 (24%) | 12 (22%) | 5 (9%) | 7 (13%) | 0.05 |

The data comprise raw test outcomes and are presented as mean (standard deviation) or n (%). P values are obtained from Generalized Estimating Equations analysis, a P value less than 0.00556 was considered statistically significant and is indicated in bold. *FVC* Forced Vital Capacity, *LLN* Lower Limit of Normal, *FEV_1_* Forced Expiratory Volume in 1s, *DLCOc* Diffusing Capacity of the Lung for Carbon monoxide adjusted for hemoglobin, *GGO* Ground-Glass Opacity

**CO-FLOW Collaboration Group**

(listed in alphabetical order)

Joachim G.J.V. Aerts^1^, L. Martine Bek^2^, Julia C. Berentschot^1^, Rita J.G. van den Berg-Emons^2^, Sieshem Bindraban^3^, Wouter J.B. Blox^4^, Jasper van Bommel^5^, Shai A. Gajadin^6^, Michel E. van Genderen^5^, Diederik A.M.P.J. Gommers^5^, Majanka H. Heijenbrok-Kal^2,7^, Merel E. Hellemons^1^, Roxane Heller^8^, Erwin Ista^9,10^, Stephanie van Loon-Kooij^11^, Chantal Luijkx^7^, Rutger Osterthun^2,7^, Laurien Oswald^3^, Gerard M. Ribbers^2,7^, Ronald N. van Rossem^11^, Herbert J. van de Sande^12^, Robert van der Stoep^13^, Janette J. Tazmi-Staal^14^, Markus P.J.M. Wijffels^7^, Eva G. Willems^14^

*Affiliations:*

^1^Department of Respiratory Medicine, Erasmus MC, University Medical Center Rotterdam, The Netherlands. ^2^Department of Rehabilitation Medicine, Erasmus MC, University Medical Center Rotterdam, The Netherlands. ^3^Department of Respiratory Medicine, Franciscus Gasthuis & Vlietland, Rotterdam, The Netherlands. ^4^Department of Respiratory Medicine, Albert Schweitzer Hospital, Dordrecht, The Netherlands. ^5^Department of Adult Intensive Care Medicine, Erasmus MC, University Medical Center Rotterdam, The Netherlands. ^6^Department of Respiratory Medicine, IJsselland Hospital, Capelle aan de IJssel, The Netherlands. ^7^Rijndam Rehabilitation, Rotterdam, The Netherlands. ^8^Department of Respiratory Medicine, Ikazia Hospital, Rotterdam, The Netherlands. ^9^Departments of Pediatrics and Pediatric Surgery, Intensive Care Unit, Erasmus MC Sophia Children's Hospital Rotterdam, The Netherlands. ^10^Department of Internal Medicine, section Nursing Science, Erasmus MC, Erasmus University Medical Center Rotterdam, The Netherlands. ^11^Department of Respiratory Medicine, Reinier de Graaf Gasthuis, Delft, The Netherlands. ^12^Aafje Nursing Home, Rotterdam, The Netherlands. ^13^Department of Physical Therapy, Erasmus MC, University Medical Center Rotterdam, The Netherlands. ^14^Laurens Intermezzo, Rotterdam, The Netherlands.
